# Genome-scale analysis of regulatory protein acetylation enzymes from photosynthetic eukaryotes

**DOI:** 10.1186/s12864-017-3894-0

**Published:** 2017-07-05

**Authors:** R. Glen Uhrig, Pascal Schläpfer, Devang Mehta, Matthias Hirsch-Hoffmann, Wilhelm Gruissem

**Affiliations:** 1 Department of Biology, Institute of Molecular Plant Biology, ETH Zurich, Universitätstrasse 2, 8092 Zurich, Switzerland; 2 Plant Biology Department, Carnegie Institution for Science, Stanford, CA 94305 USA

**Keywords:** Protein Acetylation, Photosynthetic eukaryotes, Post-translational modifications, Lysine Acetyltransferase, Lysine Deacetylase, Genomics

## Abstract

**Background:**

Reversible protein acetylation occurring on Lys-N^e^ has emerged as a key regulatory post-translational modification in eukaryotes. It is mediated by two groups of enzymes: lysine acetyltransferases (KATs) and lysine deacetylases (KDACs) that catalyze the addition and removal of acetyl groups from target proteins. Estimates indicate that protein acetylation is second to protein phosphorylation in abundance, with thousands of acetylated sites now identified in different subcellular compartments. Considering the important regulatory role of protein phosphorylation, elucidating the diversity of KATs and KDACs across photosynthetic eukaryotes is essential in furthering our understanding of the impact of reversible protein acetylation on plant cell processes.

**Results:**

We report a genome-scale analysis of lysine acetyltransferase (KAT)- and lysine deacetylase (KDAC)-families from 53 photosynthetic eukaryotes. KAT and KDAC orthologs were identified in sequenced genomes ranging from glaucophytes and algae to land plants and then analyzed for evolutionary relationships. Based on consensus molecular phylogenetic and subcellular localization data we found new sub-classes of enzymes in established KAT- and KDAC-families. Specifically, we identified a non-photosynthetic origin of the HD-tuin family KDACs, a new monocot-specific Class I HDA-family sub-class, and a phylogenetically distinct Class II algal/heterokont sub-class which maintains an ankyrin domain not conserved in land plant Class II KDACs. Protein structure analysis showed that HDA- and SRT-KDACs exist as bare catalytic subunits with highly conserved median protein length, while all KATs maintained auxiliary domains, with CBP- and TAF_II_250-KATs displaying protein domain gain and loss over the course of photosynthetic eukaryote evolution in addition to variable protein length. Lastly, promoter element enrichment analyses across species revealed conserved cis-regulatory sequences that support KAT and KDAC involvement in the regulation of plant development, cold/drought stress response, as well as cellular processes such as the circadian clock.

**Conclusions:**

Our results reveal new evolutionary, structural, and biological insights into the KAT- and KDAC-families of photosynthetic eukaryotes, including evolutionary parallels to protein kinases and protein phosphatases. Further, we provide a comprehensive annotation framework through our extensive phylogenetic analysis, from which future research investigating aspects of protein acetylation in plants can use to position new findings in a broader context.

**Electronic supplementary material:**

The online version of this article (doi:10.1186/s12864-017-3894-0) contains supplementary material, which is available to authorized users.

## Background

Reversible protein acetylation of Lys-N^ε^ is now recognized as a key regulatory post-translational modification in eukaryotic organisms, including *H. sapiens* [1], *D. melanogaster* [2], *S. cerevisiae* [3] and *A. thaliana* [4, 5]. Reversible protein acetylation is mediated by two groups of enzymes: lysine acetyltransferases (KATs) and lysine deacetylases (KDACs), which catalyze the addition and removal of acetyl groups from target proteins. Estimates indicate that protein acetylation is second to protein phosphorylation in abundance, with thousands of sites now identified in multiple subcellular compartments [6, 7].

All known eukaryotic genomes encode at least four families of KATs (MOZ, YBF2, SAS2, and TIP60 (MYST); GCN5/PCAF-related N-acetyltransferases (GNAT); p300/CREB binding protein (CBP); TATA binding protein-associated factors (TAF_II_250)) and two families of KDACs (histone deacetylase (HDA/RPD3); sirtuin (SRT)), while land plants maintain an additional family of KDACs (HD2-tuin (HDT)). Those KAT and KDAC proteins which have been characterized have been found to be primarily localized to the nucleus and cytosol [8–11], with some SRT-KDACs targeted to the mitochondria [12]. Some KDACs have also shown stimuli-dependent movement between compartments [8], which likely contributes to the diversity of subcellular compartments and protein targets for regulatory acetylation events.

In photosynthetic eukaryotes most research examining reversible protein acetylation and the corresponding KATs and KDACs has been conducted in *Arabidopsis thaliana* (Arabidopsis) [4, 5]. However, with the increased availability of sequenced genomes, analysis of protein acetylation in crop plants such as rice [13, 14], soybean [15] and grape [16] amongst others [17, 18] has been conducted. KATs and KDACs have been implicated in a number of regulatory functions. MYST-family proteins have roles in seed [19] and gametophyte [20] development as well as flowering [21], while GNAT-family proteins function in plant immunity [22], hormone signaling [23] and light signaling [24]. Less is known about the function of CBP- and TAF_II_250-family KATs in photosynthetic eukaryotes. CBP KATs are involved in the regulation of flowering [25], sugar responses [26] and ethylene signaling [27], while TAF_II_250 KATs have so far only been implicated in seed development [28]. Similarly, many KDACs have also been examined. HDA KDACs have been implicated in regulating flowering [29], gametophyte development [30], light signaling [31], cell differentiation [32, 33], seed maturation [34] and hormone signaling [35]. SRT-family proteins participate in the regulation of mitochondrial energy metabolism and metabolite transport [12], while the function of HDT-family KDACs remains largely unknown [36].

Despite an expanding volume of research investigating the roles of KDACs and KATs in photosynthetic eukaryotes, a multi-genome scale comparison is still lacking. Multi-genome scale analyses are useful in drawing molecular evolutionary connections between organisms and developing new hypotheses for protein family evolution. While molecular research is underway in photosynthetic eukaryotes ranging from algae for biofuel production [37] to rice for nutritional enhancement [38], comparative genome scale analyses provide new fundamental insights into the evolution of eukaryotic genomes and can help to discover new, conserved targets for biotechnology. The genome-scale molecular phylogenetic analysis of regulatory protein acetylation enzymes we report here demonstrates how these enzyme families have evolved in photosynthetic eukaryotes. We have found considerable changes in encoded protein complements, subcellular localization as well as protein domain organization and structure. We also identified new and unique algae-specific enzyme classes and sub-classes. Together, we have built a compendium of protein acetylation enzymes from sequenced photosynthetic eukaryotes utilizing accepted non-photosynthetic eukaryote gene and protein nomenclature to establish a clear family, class and sub-class annotation structure for photosynthetic eukaryotes.

## Results

### Prevalence of reversible protein acetylation enzymes differs in photosynthetic eukaryotes

**Fig. 1:**
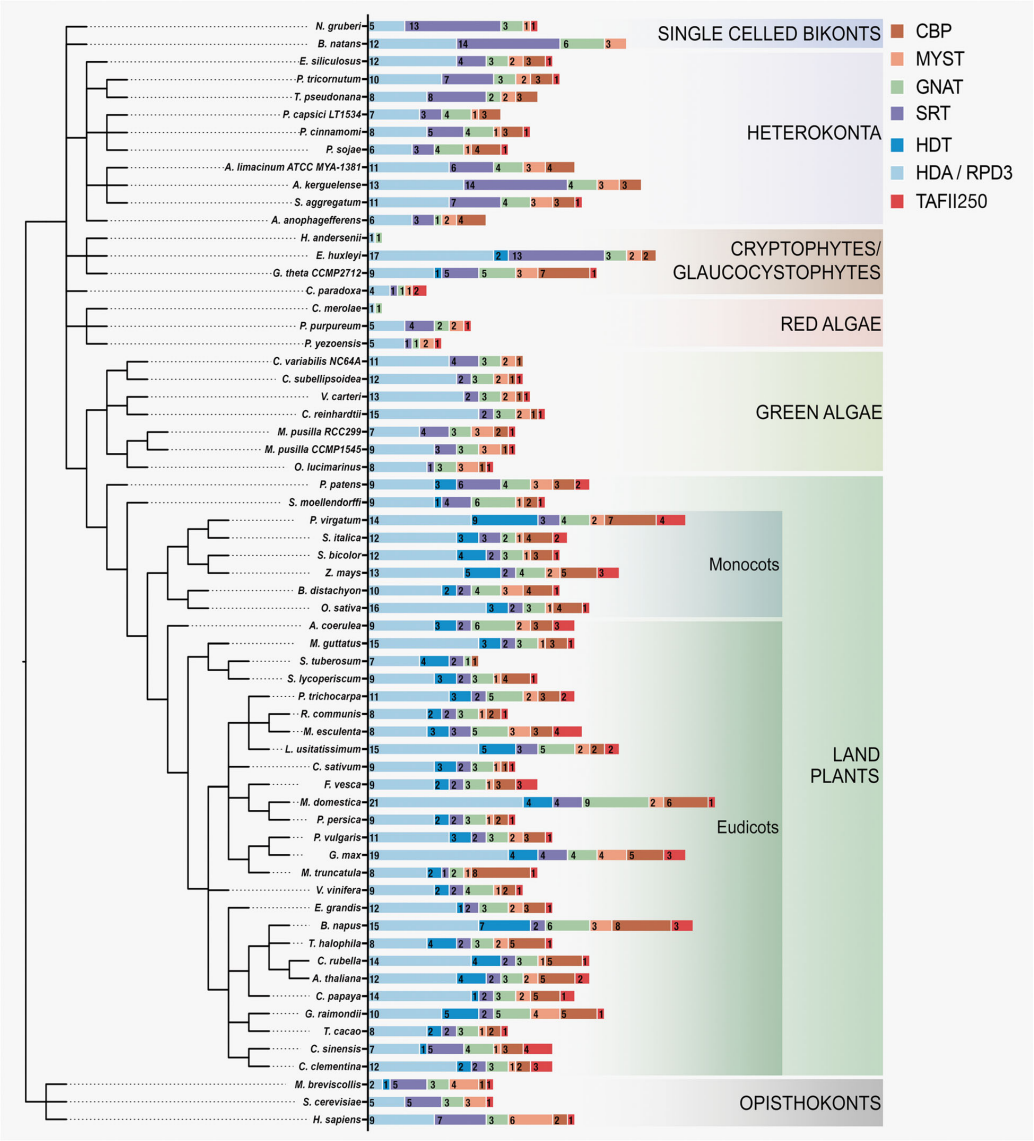
Prevalence of reversible lysine deacetylase (KDAC) and acetyltransferase (KAT) machinery across photosynthetic eukaryotes. Depicted are the histone deacetylase/reduced potassium deficiency 3 (HDA/RPD3; Light Blue), HD-tuin (HDT; Dark Blue) and sirtuin (SRT; Purple) KDACs as well as GCN5-related n-terminal acetyltransferases (GNAT; Green), MOZ, Ybf2, Sas2 and Tip60 (MYST; orange), p300/CREB binding protein (CBP; brown) and TATA binding protein associated factors (TAF_II_250; Red) acetyltransferases. The taxonomic tree was constructed using phyloT as outlined in the Materials and Methods. Each number corresponds to the number of genes encoded by that organism for the given KDAC or KAT family

Based on publically available genome resources, KDAC and KAT protein orthologs were isolated from 53 sequenced photosynthetic eukaryotes (see material and methods). Consistent with non-photosynthetic eukaryotes, we identified two KDAC and four KAT families, in addition to the previously identified plant-specific HDT KDAC-family (Fig. 1). KDAC and KAT family sizes were generally similar in land plants and comparable between monocots and dicots (Additional file 1). However, we found gene expansions and losses of specific families/members over evolutionary time. For example, monocots possessed higher average HDA, HDT and CBP protein numbers than dicots, while early land plants *S. moellendorfii* and *P. patens* maintained fewer HDA, HDT and CBP proteins but an increase in SRT and GNAT family sizes (Additional file 1).

In addition of the differences in higher plants, algae genomes have an elevated diversity in both KDAC and KAT families (Fig. 1). We found that green, red and brown algae all lack HDT KDACs, while red algae also lack CBP KATs. Furthermore, 33% of red algae exclusively maintain only HDA KDACs and GNAT KATs. Consistent with red algae, the glaucophyte *C. paradoxa* lacks CBP KATs, and consistent with all other algae, *C. paradoxa* also lacks HDT KDACs.

When we compared all photosynthetic eukaryote KDACs and KATs with representative orthologs of non-photosynthetic organisms, we discovered significant changes in the number of reversible protein acetylation enzymes in the kingdom *Plantae* (Additional file 2: Figure S1); most changes can be found among KDACs, fewer among the KATs (Additional file 2: Figure S1). HDA and HDT KDACs of both monocots and dicots have significantly expanded families, while the SRT KDACs are significantly contracted (Additional file 2: Figure S1). Similarly, monocot and dicot KDAC families also have a significant expansion (Kruskal-Wallis Test *pval* < 0.05) in HDA and HDT family sizes relative to algae (Additional file 2: Figure S2). This expansion is mirrored in the CBP-family KATs, the only KAT family that maintains a significant difference in family size with an increase in monocot and dicots relative to representative non-photosynthetic eukaryotes (Additional file 2: Figure S1). Further comparisons of photosynthetic eukaryotes revealed significant changes amongst CBP and TAF_II_250 KATs between algae and land plants, with both monocots and dicots maintaining increased family sizes (Additional file 2: Figure S3).

### Orthologous reversible protein acetylation enzymes in photosynthetic and non-photosynthetic eukaryotes have variable domain architecture

**Fig. 2:**
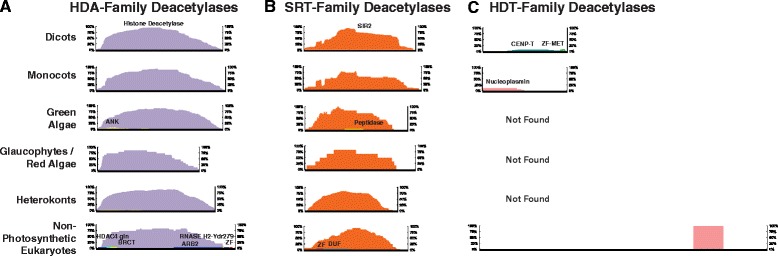
Protein sizes and functional domains of photosynthetic eukaryotic KDACs: **a**) HDA-Family Deacetylases, **b**) SRT-Family Deacetylases and **c**) HDT-Family Deacetylases. Each KDAC family was divided by species type. These divisions include: dicot, monocot, green algae, red algae/glaucophytes, heterokont/other photosynthetic eukaryotes (cryptophytes & haptophytes) and non-photosynthetic eukaryotes. Horizontal black bars depict median protein length within a given species-type for a given KDAC-family. Protein domains are depicted by colored histograms (x-axis: relative positional distribution of protein domains per depicted class of proteins, y-axis: prevalence of domain across the class of proteins). Domain information was derived from PFAM and ProSITE (Additional file 3)

**Fig. 3:**
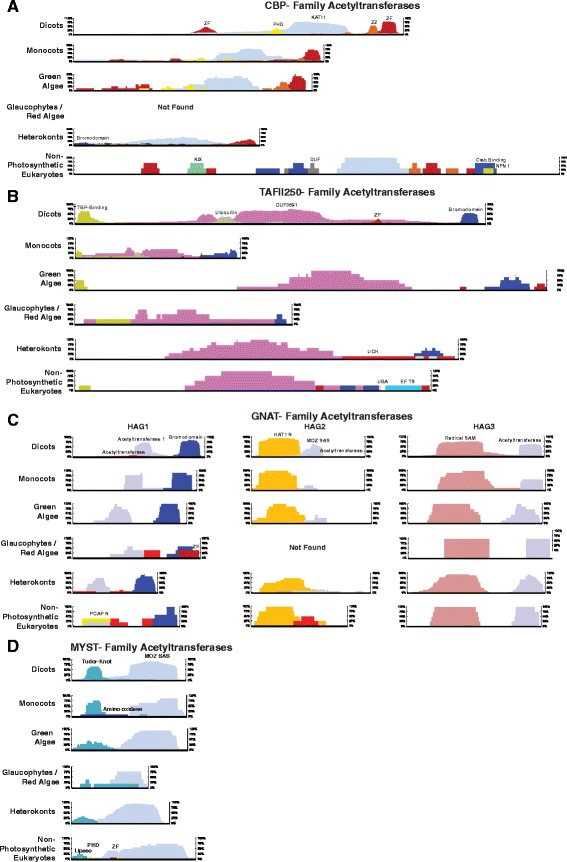
Protein sizes and functional domains of photosynthetic eukaryotic KATs: **a**) CBP-Family Acetyltransferases, **b**) TAFII250-Family Acetyltransferases, **c**) GNAT-Family Acetyltransferases, **d**) MYST-Family Acetyltransferases. Each KAT family was divided by species type. These divisions include: dicot, monocot, green algae, red algae/glaucophytes, heterokont/other photosynthetic eukaryotes (cryptophytes & haptophytes) and non-photosynthetic eukaryotes. Horizontal black bars depict median protein length within a given species-type for a given KAT-family. Protein domain are depicted by colored histograms (x-axis: relative positional distribution of protein domains per depicted class of proteins, y-axis: prevalence of domain across the class of proteins)Domain information was derived from PFAM and ProSITE (Additional file 3)

Given that the presence or absence of domains can determine protein function, we investigated the domain makeup of each KDAC and KAT family. We compared median protein size (Figs. 2 and 3, black horizontal bars), protein domain localization and protein domain distribution (Figs. 2 and 3, histograms in color; see materials and methods) for each KDAC and KAT family. With protein number and size distribution not necessarily normally distributed, median protein length was used. In photosynthetic eukaryotes protein length variation was limited for all KDAC families except the HDT KDACs (Fig. 2) while protein length was variable for 2 families of KATs; CBP and TAF_II_250 (Fig. 3). CBP KAT median protein length increased from green algae to dicots, while TAF_II_250 KATs have a variable median protein length (Fig. 3). When compared to non-photosynthetic eukaryote orthologs, median protein length of each KDAC and KAT family was comparable, with the exception of monocot CBP and TAF_II_250 KATs, glaucophyte/red algal TAF_II_250 KATs, as well as green algal and heterokont CBP KATs (Figs. 2 and 3).

We also analyzed all KDAC and KAT ortholog protein domains using PFAM and PROSITE (Additional file 3). Each KAT family possessed at least one classifiable accessory domain, while each KDAC family consists largely of only catalytic domains (Fig. 3). Protein domain locations and composition between the different photosynthetic eukaryote species were largely conserved (e.g. no domain swapping), while key differences in KDAC and KAT domain composition between photosynthetic and non-photosynthetic eukaryotes emerged. Notably, we found differences in both green algae HDA and SRT KDACs, which have subsets of proteins (<5% of sequences analyzed) with ankyrin and peptidase auxiliary domains, respectively (Fig. 2; Additional file 3). Conversely, non-photosynthetic eukaryote HDA and SRT KDACs have an array of weakly conserved auxiliary domains (threshold >5% of proteins) including BRCT, ZF, DUF and RNASE domains (Fig. 2). HDT KDACs however, encode immense accessory domain diversity, with very few domains found in greater than 5% of the HDT proteins analyzed. This included nucleoplasmin domains in monocots and CENP-T/ZF domains in dicots.

In the photosynthetic eukaryote KATs domain differences across respective orthologs relative to non-photosynthetic eukaryotes include: (1) an N-terminal bromodomain in heterokont CBP KATs (Fig. 3a), (2) the acquisition of an ubiquitin-binding domain in monocot and dicot TAF_II_250 KATs (Fig. 3b), and (3) a MOZ_SAS domain exclusively in Class II (HAG2) dicot GNAT KATs (Fig. 3c). Conversely, non-photosynthetic eukaryote KATs have several highly conserved domains absent in photosynthetic eukaryotes. These include: (1) CBP N-terminal KIX and C-terminal Creb-binding domains (Fig. 3a), and (2) MYST ligase, PHD and ZF domains (Fig. 3d).

### Gene expression and promoter elements of *KDAC* and *KAT* family members are conserved in Arabidopsis, poplar and rice

To extend the analysis of photosynthetic eukaryote KDAC and KAT conservation, we examined the developmental and stress-induced expression of Arabidopsis, *P. trichocarpa* (poplar) and *O. sativa* (rice) *KDAC*- and *KAT*-family orthologs in equivalent tissue types using ExpressoLog (http://bar.utoronto.ca/expressolog_treeviewer; [39]) (Additional file 2: Figure S4). ExpressoLog utilizes gene expression data from published microarray datasets to provide a comparative gene expression analysis between relative orthologs across plants [39]. Using Arabidopsis gene identifiers, the ExpressoLog tool provided us with correlative scoring (Spearman’s correlation coefficient; SCC) of class-specific Arabidopsis, poplar and rice *KAT* and *KDAC*-family ortholog gene expression during plant development and under stress. Class-specific Arabidopsis, poplar and rice *KAT* and *KDAC*-family ortholog expression during plant development was high (SCC = 0.8–1.0) except Class-I GNAT KATs (HAG1; GCN5-like; SCC = −0.6 to 0.0), suggesting a general functional conservation of KDAC and KAT protein orthologs across species (Additional file 2: Figure S4).

**Fig. 4:**
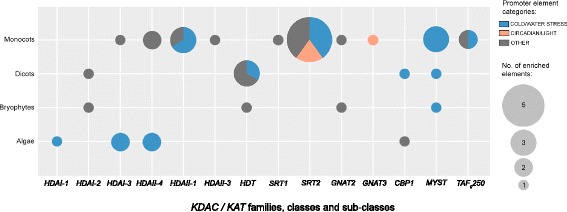
Enriched promoter elements in photosynthetic eukaryote KDAC and KAT gene families. Select dicots, monocots, moss and green algae (Additional file 4) were subjected to a promoter enrichment analysis as described in the Materials and Methods. Depicted are the KDAC- and KAT-families and the corresponding species- types that have significantly enriched promoter elements. Only those species-types and KDAC−/KAT-families maintaining an enriched promoter elements are depicted. Nomenclature used here describes protein family (e.g. HDA), class (e.g. I or 1) and sub-class (e.g. -3). TAFII250 represent the only exception to this nomenclature system

Next, we investigated the promoter regions of all *KDAC* and *KAT*-family genes for conserved cis-regulatory promoter elements in representative plants and algae using hypergeometric testing (Fig. 4). The identified conserved promoter elements were grouped into three categories based on functional regulation: cold/drought stress, light/circadian clock, and an additional category comprised of other or less-well characterized elements (Fig. 4; Additional file 4). Of these three groups, cold/drought stress-related elements were abundant and predominately comprised of ABRE-binding elements (Fig. 4).

### Molecular phylogenetic and in silico subcellular localization analyses reveal new features of photosynthetic eukaryote KDACs and KATs

#### Lysine deacetylases (KDACs)

**Fig. 5:**
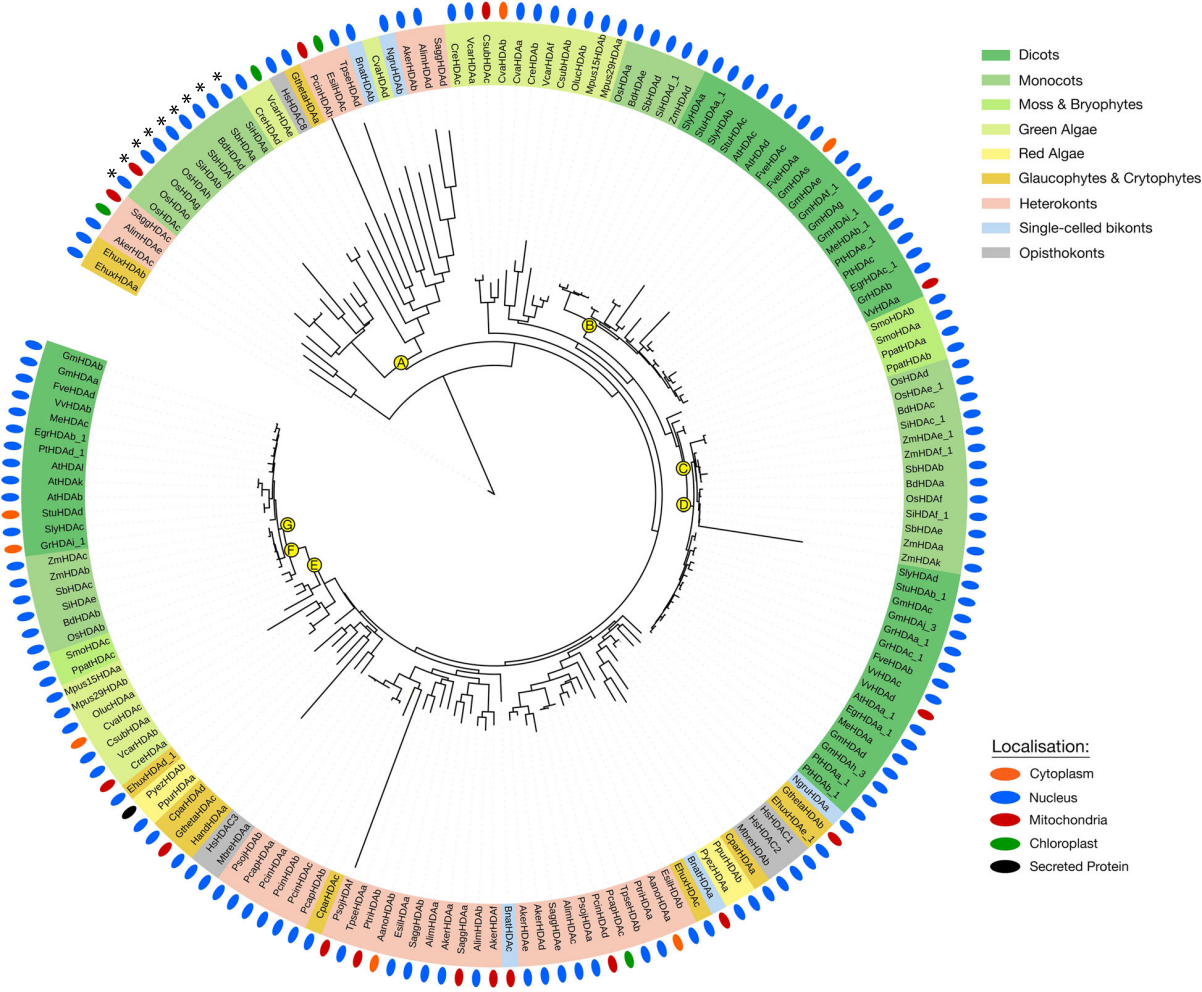
Consensus phylogenetic tree and subcellular localization information for Class I HDA-family KDACs from across photosynthetic and select non-photosynthetic eukaryotes. Phylogenetic tree inference and subcellular localization information was performed as outlined in the Materials and Methods. Key nodes are labelled with branch support values from 2 phylogenetic inference programs: PhyML and PhyloBayes. Node A: (1.0/0.80); Node B: (1.0/0.79); Node C: (0.95/0.75); Node D: (1.0/0.75); Node E: (0.99/0.89); Node F: (0.89/0.97); Node G: (0.89/0.97). Consensus subcellular localization information was derived from 5 prediction algorithms. Different species types and subcellular localizations are shown. Proteins without a known localization have no demarcation. Stars (*) denote proteins part of a monocot only Class 1 HDA sub-class. All sequences used in phylogenetic tree generation are listed in Additional file 3, while compiled in silico subcellular localization data can be found in Additional file 5

**Fig. 6:**
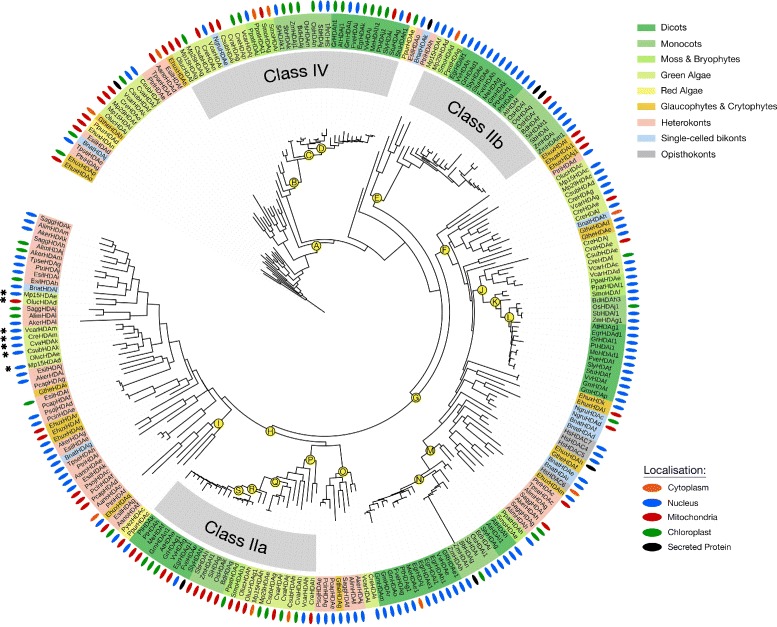
Consensus phylogenetic tree and subcellular localization information for Class II and IV HDA-family KDACs from across photosynthetic and select non-photosynthetic eukaryotes. Phylogenetic tree inference and subcellular localization information was performed as outlined in the Materials and Methods. Key nodes are labelled with branch support values from 2 phylogenetic inference programs: PhyML and PhyloBayes. Node A: (0.94/0.99); Node B: (0.86/0.99); Node C: (1.0/0.95); Node D: (0.98/0.96); Node E: (0.95/0.99); Node F: (0.60/0.72); Node G: (0.95/0.99); Node H: (0.93/0.71); Node I: (1.0/0.97); Node J: (0.99/0.80); Node K: (1.0/1.0); Node L: (1.0/1.0); Node M: (0.97/0.95); Node N: (0.83/0.90); Node O: (1.0/0.83); Node P: (1.0/0.97); Node Q: (0.99/1.0); Node R: (0.93/0.99); Node S: (0.89/0.82). Consensus subcellular localization information was derived from 5 prediction algorithms. Different species types and subcellular localizations are shown. Proteins without a known localization have no demarcation. Stars (*) denote a sub-class of green algae/heterokont HDA-family KDACs possessing an ankyrin domain. All sequences used in phylogenetic tree generation are listed in Additional file 3, while compiled in silico subcellular localization data can be found in Additional file 5

There are three families of KDACs in most photosynthetic eukaryotes. The HDA, HDT and SRT-family KDACs (Figs. 5 and 6; Additional file 2: Figure S5). HDA and SRT KDACs are found across all photosynthetic eukaryotes, while the HDT KDACs are only found in land plants (Fig. 1; Additional file 1). Consistent with their conserved role in plant development across species, the observed increase in encoded HDA KDACs parallels elevated plant complexity. Conversely, the diversity of encoded SRT KDACs decreases as plant complexity increases, with monocots and dicots lacking Class I and III SRT KDACs (Fig. 8).

#### HDA-family KDACs

HDA KDACs represent the most abundant KDACs encoded across photosynthetic eukaryotes. The red algae *C. merolae* and cryptophyte *H. andersenii* have the fewest HDA KDACs, while *M. domestica* has the most HDA KDACs (21 total). Green algae and dicots have an average of 11 HDA KDACs, while monocots average 13 (Additional file 1; Additional file 2: Figure S1). Heterokonts and representative non-photosynthetic eukaryotes have smaller numbers of HDA KDACs relative to land plants, averaging nine and five, respectively. (Additional file 1: Additional file 2: Figure S1). We found that most HDA KDACs are localized to the nucleus and cytosol, consistent with their originally described function as histone deacetylases (Figs. 5 and 6; Additional file 5); however, Class IV AtHDA2 (AtHDAj) and Class II AtHDA14 (AtHDAe) orthologs were found in different subcellular localizations between species (Fig. 6; Additional file 5). Many AtHDA2 and AtHDA14 orthologs are predicted to be localized to either the chloroplast and/or mitochondria (Fig. 6; Additional file 5).

The previously defined classes of HDA KDACs are highly conserved in all the photosynthetic eukaryotes (Figs. 5 and 6; [40]). *H. sapiens* (human) HDA KDACs (HsHDACs) were used in phylogenetic tree construction as a KDAC Class reference for the various classes of photosynthetic eukaryote HDA KDACs. This included HsHDAC1, 2, 3, and 8 (Class I), HsHDAC4, 5, and 7 (Class IIa) and HsHDAC6 (Class IIb) [41]. In our phylogenetic analyses, we discovered that all land plant HDA KDAC classes were derived from a green algal predecessor (Figs. 5 and 6). In land plants, each class of HDA KDACs consistently segregated by monocots and dicots, with the origins of each class anchored by the early land plants *S. moellendorfii* and *P. patens*. The higher number of monocot HDA KDACs (approximately one additional member) results from a new green algae-derived cytosolic Class I HDA KDAC family, which is exclusive to monocots (Fig. 5, node A). Interestingly, this class is absent in *Z. mays* (maize) and *P. virgatum* (Switchgrass), but is particularly expanded in rice with four members (Fig. 5 node A).

Despite family members clearly clustering as part of the Class I, II and IV, we could not identify a distinct Class IIa and b division. Furthermore, we found algae to have a number of divergent HDA KDACs basal to distinct algae/heterokont-specific HDA KDAC protein sub-classes. For example, algae and heterokonts form divergent Class II and IV HDA KDAC sub-classes that are absent in land plants (Fig. 6). Both these sub-classes were derived from either red or green algae ancestors, with the Class IV sub-class predominantly localized to the mitochondria and the Class II sub-class localized to the nucleus (Fig. 6). Overall, green algae and heterokonts maintain higher HDA KDAC diversity relative to land plants.

#### HDT-family deacetylases

**Fig. 7:**
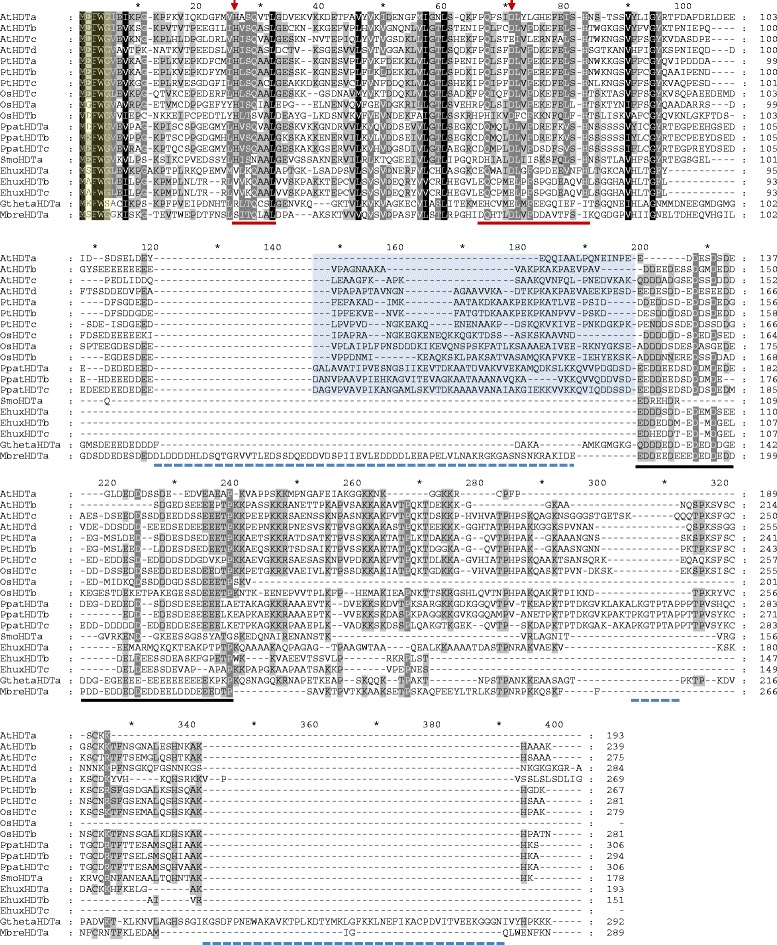
Alignment of Select HDT-family KDACs from photosynthetic eukaryotes. Representative dicots (*A. thaliana* & *P. trichocarpa*), monocots (*O. sativa*), moss (*P. patens*), bryophytes (*S. moellendorfii*) and chromoalveolates (*E. huxleyi* and *G. theta*) are shown. The short half-life motif is conserved in the extreme n-terminus of all HDT KDACs (yellow) along with the first 100 amino acids of each HDT KDAC. Stretches of unique protein regions can be observed in *M. brevicolis*, *G. theta* and *P. patens* HDT KDACs (blue dashes). As well, absence of conserved land plant protein regions is also observed (shaded blue). Arrows (red) denote amino acids involved in HDT deacetylase activity. Underlined (solid red) are the larger proposed catalytic motifs. A highly conserved acidic amino acid stretch is also depicted (Underlined; solid black). *M. brevecolis* maintains a large n-terminal region not conserved amongst other HDT KDACs (not shown). This may be an unannotated splice variant or a mis-annotation in the *M. brevicolis* genome. The n-terminal methionine depicted here was highly conserved across all HDT-family KDACs found in this study

HDT KDACs are found primarily in land plants, in which a discernible phylogenetic separation was found between monocots and dicots. Both *S. moellendorfii* and *P. patens* have HDT KDACs; however, we were not able to find HDT KDACs in glaucophytes, green, red, or brown algae (Additional file 2; Figure S5). This is consistent with other findings [42], which proposed that HDT KDACs are exclusive to land plants [36, 40, 42]. Contrastingly, our findings suggest a more ancient origin, as an HDT-family ortholog was found in the distant metazoan ancestor *M. brevicolis* (Fig. 7; Additional file 2: Figure S5). Similar to HDA KDACs, most HDT KDACs have a predicted nuclear and/or cytosolic localization, including the *M. brevicolis* ortholog (MbreHDTa) [43]. Oddly, HDT KDACs from *E. huxleyi* and *G. theta* have a predicted mitochondrial localization (Additional file 2; Figure S5, Additional file 5).

#### Sirtuin (SRT)-family deacetylases

**Fig. 8:**
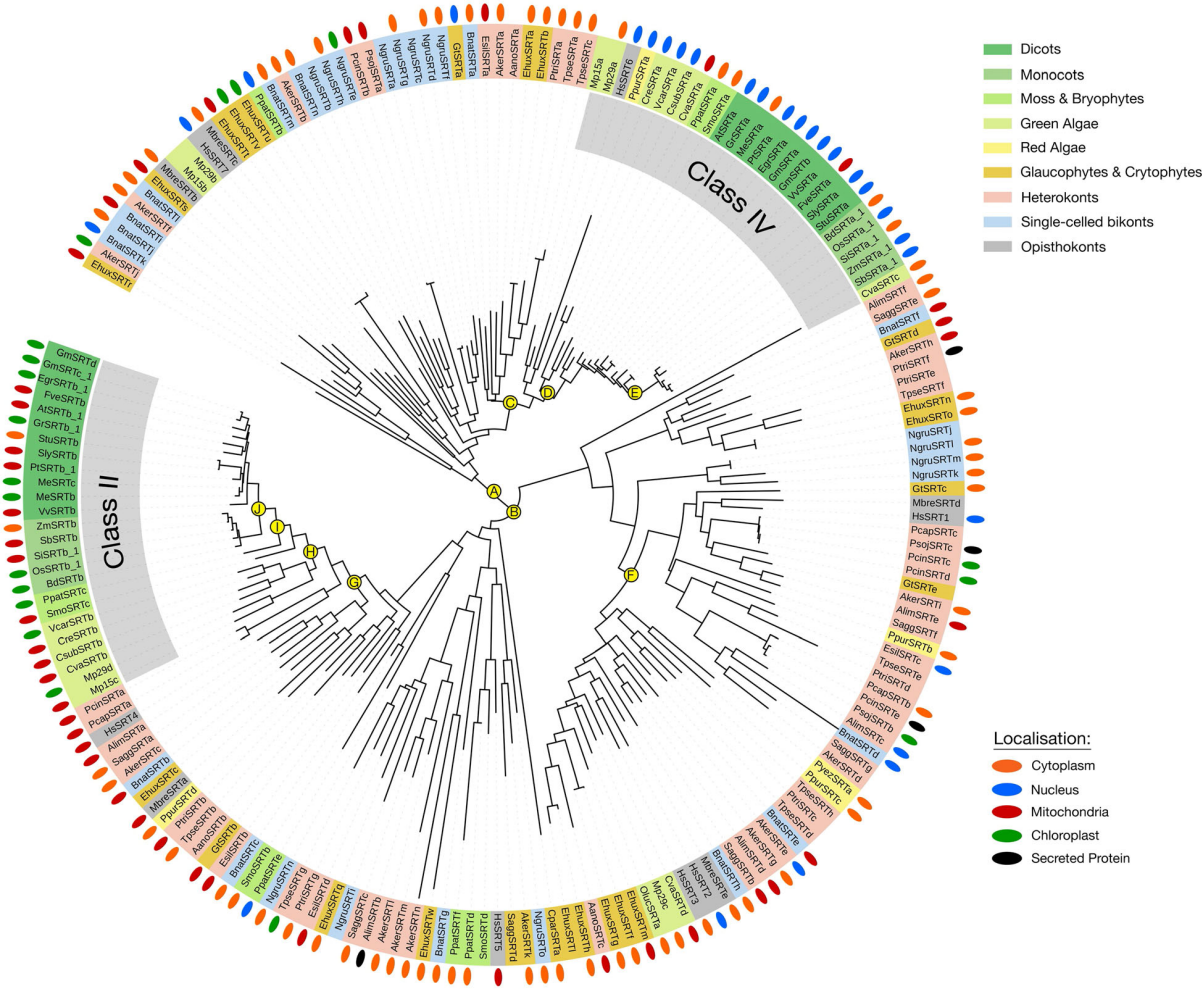
Consensus phylogenetic tree and subcellular localization information for SRT-family KDACs from across photosynthetic and select non-photosynthetic eukaryotes. Phylogenetic tree inference and subcellular localization information was performed as outlined in the Materials and Methods. Key nodes are labelled with branch support values from 2 phylogenetic inference programs: PhyML and PhyloBayes. Node A: (0.99/0.73); Node B: (0.99/0.97); Node C: (0.82/0.97); Node D: (0.93/0.99); Node E: (0.93/0.99); Node F: (0.94/0.99); Node G: (0.99/0.93); Node H: (0.82/0.92); Node I: (0.99/1.0); Node J: (0.79/0.75). Consensus subcellular localization information was derived from 5 prediction algorithms. Different species types and subcellular localizations are shown. Proteins without a known localization have no demarcation. All sequences used in phylogenetic tree generation are listed in Additional file 3, while compiled in silico subcellular localization data can be found in Additional file 5

SRT KDACs are lysine deacetylases that require nicotinamide adenine dinucleotide (NAD^+^) as a co-factor for catalysis. This differentiates them from the metal-dependent KDACs. There are four main classes (I – IV) of SRT KDACs in eukaryotes [40, 44]. In humans these include: HsSRT1, 2 and 3 (Class I), HsSRT4 (Class II), HsSRT5 (Class III) and HsSRT6 and 7 (Class IV) [44]. Only the Class II and IV SRT KDAC orthologs that are localized to mitochondria/plastid (Class II) and nucleus/cytosol (Class IV) are found in dicots and monocots, while Class I and III orthologs are found in an array of other photosynthetic eukaryotes (Fig. 8). Relative to the non-photosynthetic eukaryotes *M. brevicollis*, yeast and humans, which each have an average of eight SRT KDACs*,* green algae and land plants encode only two or three SRTs, while moss and bryophytes encode an average of five SRT KDACs. Conversely, rhizaria, haptophytes and heterolobosea as well as one heterokont species have significantly higher numbers of SRT KDACs encoded by 13–14 genes (Additional file 1; Additional file 2; Figure S1).

Land plant, red and green algal Class II and IV SRT KDACs consistently cluster with their human orthologs HsSRT4 (mitochondria) and HsSRT6 (cytosol/nucleus) [44], with the predicted subcellular localization of photosynthetic eukaryote Class II and IV SRT KDACs conserved (Fig. 8) [12]. Similarly, Class I HsSRTs cluster with a variety of photosynthetic eukaryote SRT KDAC orthologs independent of Class II and IV SRT KDACs (Fig. 8). The majority of these SRT KDAC orthologs have a nuclear/cytosolic subcellular localization. Basal photosynthetic eukaryote *C. paradoxa* encodes only a single SRT ortholog, which consistently clusters with the Class II SRT KDACs that are targeted to mitochondria/plastids, despite its predicted cytosolic localization.

#### Lysine acetyltransferases (KATs)

**Fig. 9:**
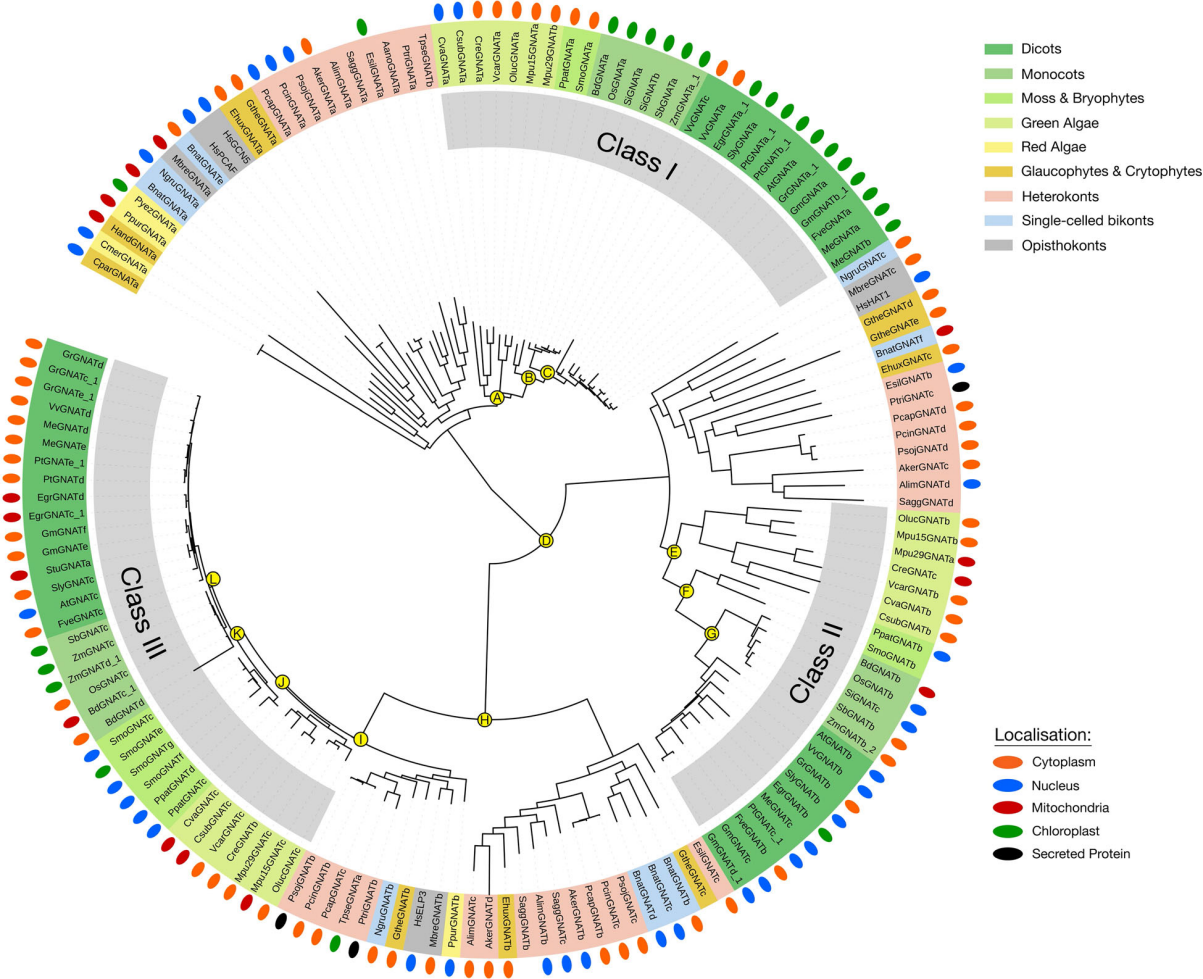
Consensus phylogenetic tree and subcellular localization information for GNAT-family KATs from across photosynthetic and select non-photosynthetic eukaryotes. Phylogenetic tree inference and subcellular localization information was performed as outlined in the Materials and Methods. Key nodes are labelled with branch support values from 2 phylogenetic inference programs: PhyML and PhyloBayes. Node A: (0.98/0.56); Node B: (0.99/0.97); Node C: (0.99/1.0); Node D: (1.0/1.0); Node E: (0.84/0.93); Node F: (0.98/0.99); Node G: (1.0/1.0); Node H: (1.0/1.0); Node I: (0.99/0.76); Node J: (0.5/0.86); Node K: (0.99/0.97); Node L: (0.94/0.97). Consensus subcellular localization information was derived from 5 prediction algorithms. Different species types and subcellular localizations are shown. Proteins without a known localization have no demarcation. All sequences used in phylogenetic tree generation are listed in Additional file 3, while compiled in silico subcellular localization data can be found in Additional file 5

**Fig. 10:**
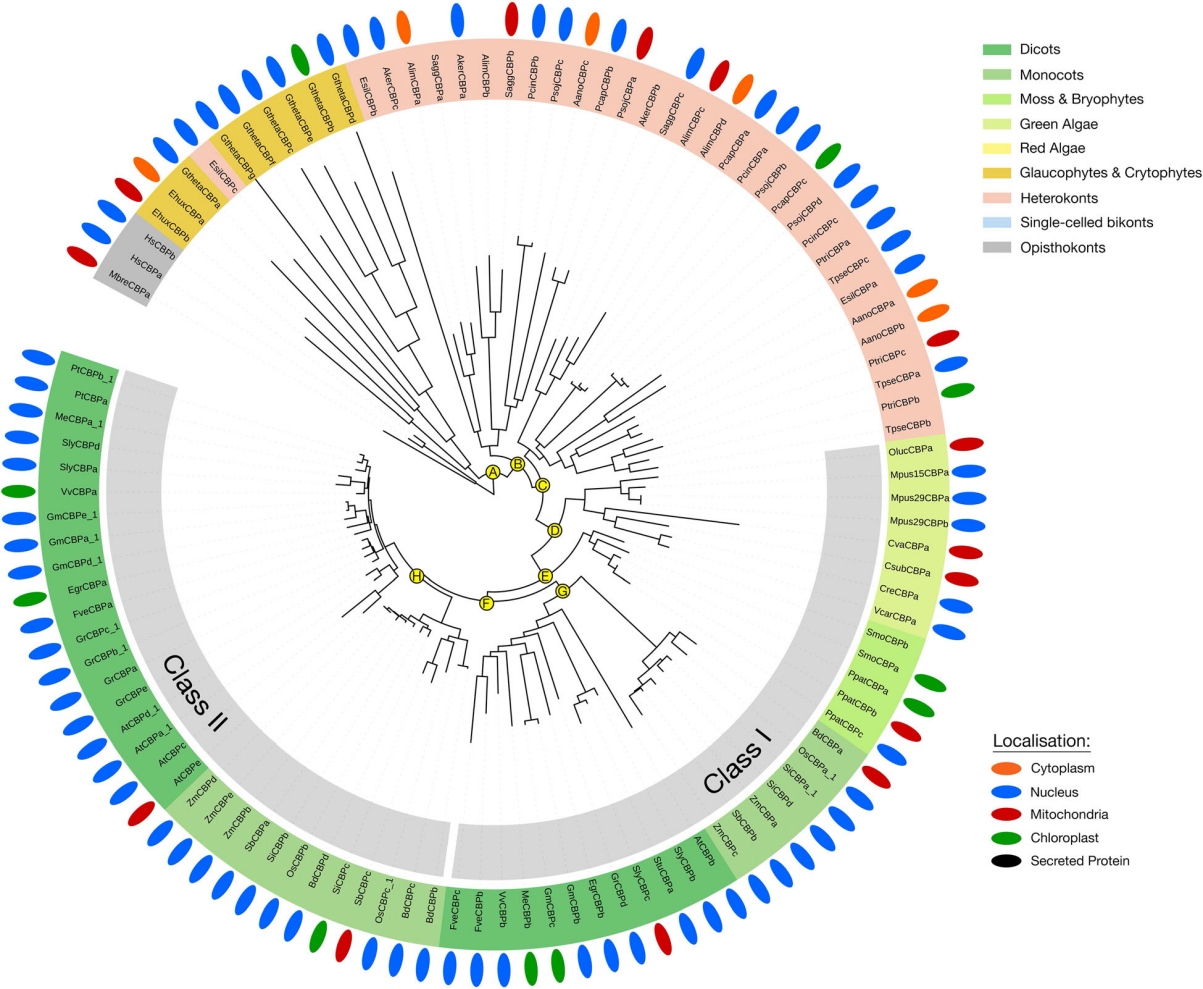
Consensus phylogenetic tree and subcellular localization information for CBP-family KATs from across photosynthetic and select non-photosynthetic eukaryotes. Phylogenetic tree inference and subcellular localization information was performed as outlined in the Materials and Methods. Key nodes are labelled with branch support values from 2 phylogenetic inference programs: PhyML and PhyloBayes. Node A: (1.0/1.0); Node B: (0.87/0.91); Node C: (0.86/0.82); Node D: (1.0/1.0); Node E: (1.0/1.0); Node F: (0.95/1.0); Node G: (0.97/1.0); Node H: (0.6/0.53). Consensus subcellular localization information was derived from 5 prediction algorithms. Different species types and subcellular localizations are shown. Proteins without a known localization have no demarcation. All sequences used in phylogenetic tree generation are listed in Additional file 3, while compiled in silico subcellular localization data can be found in Additional file 5

Photosynthetic eukaryotes encode 4 families of KATs: the MYST, GNAT, TAF_II_250 and CBP-families. Unlike the KDACs, we found that all families and classes of KATs found in non-photosynthetic eukaryotes are conserved in photosynthetic eukaryotes (Fig. 1; Additional file 1). In photosynthetic eukaryotes however, two sub-classes of CBP KATs emerged which contribute to the overall increase in photosynthetic eukaryote CBP KATs, while throughout each KAT family heterokont orthologs were found to consistently diverge from those in other photosynthetic eukaryotes (Figs. 9 and 10; Additional file 2; Figure S6 and S7).

#### MYST-family acetyltransferases

MYST acetyltransferases represent a highly conserved family of protein acetylation enzymes (Additional file 2: Figure S6), with land plant MYST KATs being founded by a green algae ancestor. Green algae, heterokonts, other photosynthetic eukaryotes and land plants all encode an average of two MYST KATs, while representative non-photosynthetic eukaryotes maintained an average of four MYST KATs (Additional file 1; Additional file 2; Figure S6). The subcellular localization of MYST KATs is also conserved as most photosynthetic and non-photosynthetic orthologs have a predicted nuclear localization (Additional file 2: Figure S6). We found only one of the two green algal MYST KAT sub-classes to be basal to land plants (Additional file 2: Figure S6). As well, we found land plant MYST KATs to be encoded by closely related multi-copy families, making it likely that land plant MYSTs underwent gene duplication. This is supported by the presence of two closely related MYST KAT orthologs in *P. patens*. Furthermore, in Arabidopsis, MYST KATs have been found to be functionally redundant [20]; however, further examination in other organisms is required to confirm if this is an evolutionarily conserved phenomenon.

#### GNAT-family acetyltransferases

GNAT KATs cluster into three highly conserved protein classes in all eukaryotes. Each organism encodes a single gene copy from each of the three classes, except red algae and *C. paradoxa,* which have only one GNAT (Fig. 9). GNAT Class I, II and III represent the HAG1 (GCN5-like), HAG2 (HAT1-like) and HAG3 (ELP3-like) acetyltransferases; respectively. In photosynthetic eukaryotes, each class of GNAT KATs has a green algae origin. Phylogenetic analyses revealed distinct and diverse sub-classes (Class I - III) of GNAT KATs. These sub-classes are comprised of red algae and heterokont orthologs, while a diverse cluster consists of mainly heterokont proteins (Fig. 9). Class I GNAT KATs are predominantly predicted to be nuclear/cytosol localized in algae, but in land plants they are found exclusively in chloroplasts. Class III monocot orthologs are predicted to localize either to mitochondria or chloroplasts, while dicot orthologs are cytosol-localized. All Class II GNAT KATs have predicted nuclear and/or cytosol localizations.

#### TAF_II_250-family acetyltransferases

TAF_II_250 KATs expanded from an average of one gene in non-photosynthetic eukaryotes to two genes in monocots and dicots (Additional file 2; Figure S7). TAF_II_250 KATs are also found in red algae and *C. paradoxa*, indicating their presence in early photosynthetic eukaryotes. Many heterokonts lack a TAF_II_250 KAT, likely indicating gene loss in some organisms. Similar to MYST and CBP KATs, a general expansion of TAF_II_250 has occurred in land plants (Additional file 1; Additional file 2: Figure S7). High branch support indicates clear divisions between green algae, moss/bryophytes and higher plants, with further segregation of monocot and dicot TAF_II_250s (Additional file 2: Figure S7). Despite the expansion of TAF_II_250 KATs in some land plants, their predicted nuclear localization is highly conserved for all eukaryote TAF_II_250s with few exceptions (Additional file 2: Figure S7).

#### CBP-family acetyltransferases

CBP KATs have a complex evolutionary ancestry that has resulted in a large family expansion in land plants. They are consistently found throughout the photosynthetic eukaryotes as well as the distal non-photosynthetic eukaryote *M. brevicolis* (Fig. 10). Representative non-photosynthetic eukaryotes and green algae have one gene on average, while red algae and *C. paradoxa* encode no CBP KATs (Additional file 1; Additional file 2: Figure S1). Monocots and dicots however, encode an average of four to five CBP KATs, while moss and bryophytes have two to three. This seems to indicate ancestral gene duplication events following the colonization of land. Land plants represent the first instance of CBP segregation into Class I and II proteins (Fig. 10). Heterokonts and other photosynthetic CBP KATs reliably clustered into two classes denoted by nodes A, B and C (Fig. 10). CBP KATs have a predominantly nuclear and/or cytosol localization, although a small number of these enzymes are also predicted to localize to mitochondria or plastids.

## Discussion

### Reversible protein acetylation enzymes show parallels to reversible protein phosphorylation enzymes

Protein acetylation is an abundant protein post-translational modification (PTM) in all eukaryotes, second only to protein phosphorylation [7]. Similar to protein kinases (PKs) and phosphatases (PPases) that catalyze reversible protein phosphorylation, KATs and KDACs catalyze the addition and removal of acetyl groups from lysine residues (Lys-N^ε^) of target proteins [45]. There are several other parallels between the enzymes responsible for catalyzing reversible protein phosphorylation and acetylation that may suggest more wide-ranging evolutionary commonalties between these different regulatory PTM enzymes. For example, the four PPase families have different catalytic mechanisms ranging from diverse metal cation co-factors in PPP- (Mn^2+^) and PPM- (Mg^2+^) family PPases to cysteine and aspartate, metal-independent mechanisms in PTP and Asp-based PPases [46]. Similarly, KDACs utilize different co-factors, with HDA and SRT KDACs requiring Zn^2+^ and NAD^+^ for catalytic activity, respectively [47]. Furthermore, many PPases consist of catalytic subunits that require interaction with other proteins to achieve their specificity [48]. Accordingly, PPP-family PPases associate with hundreds of targeting subunits in mammals and other non-photosynthetic eukaryotes to determine substrate specificity [49]. Based on the compilation of KDACs (585 HDAs; 200 SRTs) from each KDAC family into representative protein models, both HDA and SRT KDACs were also consistently encoded as bare catalytic subunits in all photosynthetic eukaryotes (Fig. 2). Considering the large number of annotated proteins that are acetylated [7], it is likely that HDAs also require interactions with targeting subunits for substrate specificity. This is supported by KDAC protein interactome data from HeLa cell culture in which 200 previously uncharacterized protein complexes were found [9]. It is therefore conceivable that the HDA protein interactome in photosynthetic eukaryotes will be comparable.

Conversely, both PKs and KATs have several accessory domains that likely assist in substrate specificity. Unlike PKs however, KATs do not form a superfamily of proteins. Structural studies of non-photosynthetic eukaryote KATs indicate a diversity among the catalytic mechanisms of the MYST [50], GNAT [51] and CBP KATs [52]. Furthermore, photosynthetic and non-photosynthetic eukaryotes have fewer KATs than PKs. For example, Arabidopsis has 12 KATs compared to around 1000 PKs [53]. Considering the prevalence of protein acetylation in plants [7], this stark difference indicates that KATs likely also require different protein interaction partners to determine their substrate specificity. To date however, only a limited number of KAT protein interactome studies have been conducted in either photosynthetic or non-photosynthetic eukaryotes [54, 55]. Collectively, these commonalities suggest a form of convergent evolution amongst the enzymes responsible for catalyzing the two most prolific PTMs; phosphorylation and acetylation.

### Genome scale analyses of KDAC and KAT gene families: a new understanding of origins and protein structure in photosynthetic eukaryotes

Despite photosynthetic eukaryotes possessing many of the same KDAC- and KAT-family features as their non-photosynthetic orthologs, we have identified a number of unique features, including differences in origin and protein structure. In particular, HDT KDACs, currently classified as exclusively a land plant KDAC family, were found here to originate outside of photosynthetic eukaryotes at the base of eukaryote evolution, while the CBP KATs exhibit major protein structure differences between photosynthetic and non-photosynthetic eukaryotes indicative of a conserved substitution in protein domains.

Previous reports suggested that HDT KDACs are a plant-specific KDAC family [40, 42]. Our analysis found a distant eukaryote origin of HDT KDACs in the basal non-photosynthetic eukaryote *M. brevicollis* (MbreHDTa), indicating that HDT KDACs are in fact not plant specific. Reciprocal BlastP analysis supports MbreHDTa as a HDT family protein, while alignment of MbreHDTa to plant HDTs reveals conservation of key HDT family motifs (Fig. 7). Further analysis of algae, heterokonts and other photosynthetic eukaryotes showed that they lack HDT KDACs, except in *E. huxleyi* and *G. theta*. This suggests either loss of HDT KDACs or an independent acquisition of HDT KDACs by photosynthetic eukaryotes. Since HDT KDACs are present in *E. huxleyi* and *G. theta*, it is most likely that HDT KDACs were lost in those photosynthetic eukaryotes that lack them.

We also found HDT KDAC protein structure to be highly divergent amongst orthologs. Only few domains are present in more than 5% of the analyzed HDT KDACs, in contrast to HDA and SRT KDACs, which are highly conserved catalytic domain-only proteins (Fig. 2). Based on the distant *M. brevicollis* HDT ortholog, the nucleoplasmin histone-binding domain seems to be most conserved in all organism classes and is suggested to function as a scaffold (Fig. 2) [56]. This is also reflected in the conserved domain complement of each species class, for example the nucleoplasmin domain is conserved in monocots but absent in dicots (Fig. 2; Additional file 3). Alternatively, dicot HDTs maintain a CENP-T domain. However, despite these differences, both nucleoplasmin and CENP-T domain containing proteins function to regulate chromatin [56, 57]. To date, the function of the HDT KDACs remains largely unresolved, although roles in seed germination [58] and stress response [59] have been proposed.

The CBP KAT-family has expanded in land plants (Figs. 1 and 10), beginning in moss and bryophytes with the emergence of two evolutionary distinct and conserved clusters of CBP KATs (Fig. 10). Examination of CBP KAT domain elements revealed differences in domain complements between photosynthetic and non-photosynthetic eukaryotes. Photosynthetic eukaryote CBP KATs lack the canonical kinase-inducible KIX domain (Additional file 2: Figure S8), instead maintaining a plant homeodomain (PHD) domain at a different location in the protein. Both domains allow CBP KATs to influence gene expression, but likely in different ways. The KIX domain acts as a phosphorylation-mediated docking site for transcriptional activators allowing non-photosynthetic eukaryote CBP KATs to function in protein complexes to regulate transcription [60]. The PHD domain allows photosynthetic eukaryote CBP KATs to bind methylated histones to regulate transcription in a phosphorylation independent manner [61]. Since CBP KATs remain poorly characterized in photosynthetic eukaryotes, further examination across species required to see if functional conservation exists.

### Promoter element enrichment analysis provides new insights into KDAC and KAT function across species

Through comparative promoter element analysis, we found five cold/water stress response elements *DRE-like*, *DPBF1* and *2*, *ABRE-like*, *ABFs*, and *CBF1BS in COR15a* enriched amongst various classes and sub-classes of each KDAC and KAT family (Fig. 4). In particular, algal and monocot *HDAs*, monocot *SRT2s* as well as monocot and dicot *MYSTs*. Implication of involvement in stress response based on promoter elements was further supported by our ExpressoLog analysis for these protein classes and sub-classes in stress response (Additional file 2: Figure S4). For example, rice (monocot) *HDA* Class I-2 orthologs of AtHDAa, were found to exhibit high correlative expression across tissues under stress. Similarly, Class II *SRT* and *MYST* KDAC orthologs in rice also showed strong correlative expression under stress (Additional file 2: Figure S4). Interestingly, Class II *SRT* KDACs are the only known mitochondria-localized KDACs in photosynthetic eukaryotes and have been shown to regulate mitochondrial energy metabolism and metabolite transport [12]. The presence of cold and drought *ABRE/ABF* cis-regulatory elements in Class II *SRT* promoters may connect abiotic stress to adaptive responses requiring the adjustment of mitochondrial function. For example, oxidative phosphorylation complex I function has been shown to be modified by cold/drought [62] as well as ABA [63] and has also been shown to directly interact with SRT2 in Arabidopsis [12], indicating a potential connection between reversible protein acetylation and mitochondrial stress response.

In addition to enrichment of cold and drought stress response promoter elements, our analysis also found conservation of circadian- and light-responsive cis-regulatory elements in the promoters of monocot Class II *SRT* KDACs and Class III *GNAT* KATs. Considering the central nature of mitochondria to proper cell functionality, a connection between Class II *SRT* KDACs and light-response in plants is interesting [64]. The light-responsive promoter element *HBOXCONSENSUSPVCHS* may offer to connect Class II *SRT* KDACs to the daily fluctuations in mitochondrial metabolism [64]. Furthermore, our analysis also revealed enrichment of a CCA1-binding element [65, 66] in the promoters of Class III *GNAT* KATs (ELP3-KATs), which are nuclear/cytosolic proteins implicated in hormone signaling [23] and cell proliferation [67]. Both these processes are controlled by the circadian clock [68], indicating that reversible protein acetylation may also be a circadian controlled process.

Lastly, we resolved a number of ‘other’ enriched cis-regulatory promoter sequences including a nitrogen-responsive element (*EMHVCHORD*), a heat-stress response element (*HSEs*_binding_site_motif) and a flowering time-related *CArG* element enriched in monocot Class II-4 & -3 *HDA* and dicot *HDT* KDACs (Fig. 4). Interestingly, the Arabidopsis Class II-3 *HDA* KDAC ortholog ‘i’ has previously been characterized to be involved in Arabidopsis flowering, with *hda-i* (*hda5*) plants exhibiting delayed flowering and up-regulation of FLOWERING LOCUS (FLC) gene expression. This same study also showed a direct interaction between HDAi and transcription factors FVE and FLD [29]. With literature supporting many of the biological functions proposed by our cis-regulatory element enrichment analysis, further research should look to investigate KDAC and KAT Class and sub-class function in relation to roles identified as part of our cis-regulatory element enrichment analysis which have not yet been explored (e.g. a role for KDAC and KAT regulation of nitrogen responses).

### Algae and heterokonts: a new frontier in understanding protein acetylation?

Algae and heterokonts represent the most diverse set of photosynthetic eukaryotes. Glaucophytes, green and red algae occupy the base of kingdom Plantae, while heterokonts emerged from algae through secondary endosymbiosis. Algae are of particular interest as the progenitors of land plants, but also because of their ability to acquire new genes via horizontal gene transfer events, leading to the acquisition and adaptation of genes with new functions and/or capabilities [69–73]. In light of this we examined our phylogenetic analysis for algal KDAC and KAT families. Here we found a number of unique protein sub-classes not found in land plants, but conserved amongst algae, heterokonts and other photosynthetic eukaryotes. Of these, the HDA and SRT KDACs as well as MYST and GNAT KATs have the largest number of unique protein sub-classes.

Both HDA and SRT KDACs have at least one well supported algae-containing, non-land plant protein sub-class (Figs. 5, 6 and 8). The unique SRT KDAC class is almost exclusively comprised of heterokont orthologs, which account for the major expansion of the SRT KDACs in these organisms (Fig. 8; node F). Conversely, algae and plants have a similar number of HDA KDACs as a result of likely gene duplication within one land plant Class I HDA clade (Fig. 5) combined with the increased number of unique algae/heterokont specific sub-classes. However, despite the phylogenetic diversity of these algae/heterokont KDAC sub-classes, they have minimal differences in their protein domains, suggesting that HDA and SRT KDACs are functionally conserved among the photosynthetic eukaryotes from a structure-function perspective (Fig. 2). As an exception, some phylogenetically distinct algal Class II HDA KDACs have a highly conserved Ankyrin domain in their N-terminus (Fig. 6; node H). Ankyrin domains function as protein-protein interaction platforms and are found in a variety of protein types [74], suggesting that this cluster of algae/heterokont-specific HDA KDACs may have a unique biological functions and protein interactors.

Similarly, MYST and GNAT KATs have algae/heterokont-specific protein sub-classes, respectively (Fig. 9, Additional file 2: Figure S6). Land plants and green algae both have an average of two MYST KATs; however, all land plant MYST KDACs were derived from the duplication of single green algae protein progenitor, while a second green algae-only protein sub-class founded the heterokont MYST KDACs (Additional file 2: Figure S6 node B). We also resolved a second sub-class of heterokont MYST KATs founded by red algae (Additional file 2: Figure S6 node A). The unique algae/heterokont-specific GNAT KATs are characterized by two conserved phylogenetic divisions. The first was the formation of a red algae-containing protein sub-class (Fig. 9; node I) and the second was a distinct divergence of some heterokont Class-III GNAT (HAG3; ELP3-like) KATs (Fig. 9; node H). Since respective non-photosynthetic eukaryote orthologs of *N. greubi*, *M. brevicolis* and *H. sapiens* appear at the base of each highly conserved GNAT KAT Class (I - III), the sub-clustering of heterokont Class-III KATs may be the result of a gene duplication event. Similar to algae/heterokont-specific MYST KATs, no differences were detected in the protein domain composition of algae/heterokont-specific GNAT KATs, indicating that they have conserved structure-function (Fig. 3). With algae and heterokonts representing a frontier of natural product discovery and biofuel production [75–77] identifying and characterizing proteins such as KDACs and KATs will be fundamental in better understanding how these systems are regulated.

## Conclusions

Genome-scale molecular phylogenetic analyses facilitate the understanding of gene family conservation and reveal new information about protein divergence throughout evolution, such as the acquisition of new domains or re-targeting to new subcellular compartments. Our comprehensive analysis of the KDAC and KAT-families responsible for reversible protein acetylation from 53 photosynthetic eukaryotes provides an essential framework for future investigation of regulatory protein acetylation in plants. Our genome scale analysis has identified new structural elements central to the function of KDACs and KATs, including the identification of HDAs and SRTs as conserved bare catalytic subunits across photosynthetic eukaryotes. Furthermore, we resolved a number of new protein classes and sub-classes in well-established protein families; in particular a number of new algae/heterokont-specific protein sub-classes. Both algae and regulatory protein acetylation represent emerging frontiers in plant science research, rendering an understanding of reversible protein acetylation in algae and heterokonts of significant interest. For example, what are the function(s) of algal-specific KDAC/KAT gene families? Why are they not present in land plants? Future targeted studies should aim to address these open questions.

## Methods

### Candidate sequence isolation and validation

Protein sequences of KDAC and KAT family members were obtained from *A. thaliana*, *P. trichocarpa* (Poplar) and *O. sativa* (Rice) genomes using the BlastP option of Phytozome v9.1 (http://www.phytozome.org). Isolated protein sequences were employed in generating multiple sequence alignments as previously described [73]. KDAC families HDA and SRT were further divided into 2 and 3 separate alignments based on a previously annotated Class structure [40]. Within the four KAT families, only GNAT KATs were further sub-divided into three Classes, while all remaining KDAC and KAT families were aligned without further sub-division. Sequence alignments were then converted into Stockholm format and used to generate HMMs by HMMER (version 3.0) software [78]; http://hmmer.org/). A database of protein sequences from sequenced photosynthetic eukaryotes was complied with sequenced photosynthetic eukaryote genomes obtained from Phytozome (Version 9.1; http://www.phytozome.net/), Department of Energy Joint Genome Institute (DOE-JGI; http://www.jgi.doe.gov/) as well as individual genome project websites: *C. merolae* (http://merolae.biol.s.u-tokyo.ac.jp/); *C. paradoxa* (http://cyanophora.rutgers.edu/cyanophora/), *E. siliculosus* (http://bioinformatics.psb.ugent.be/genomes/view/Ectocarpus-siliculosus). The protein database was searched using the constructed HMMs for each protein family, with candidate sequences extracted and used in further formulation of new multiple sequence alignments as described above. HMM identified candidate orthologs from each protein family and class were filtered for a statistical threshold below e^−100^. All isolated HMM candidate sequences ranging in E-value from e^−100^ to 0.001 were manually evaluated through additional alignments and reciprocal BlastP analysis (http://blast.ncbi.nlm.nih.gov/).

### Domain analysis and consensus sequence creation

To analyze putative domains from each species type (e.g. dicots) for each KDAC and KAT family, FASTA files containing protein sequences for each KDAC and KAT-family from each species type were assembled. All protein sequences used for each representative protein model were submitted to PFAM (http://pfam.xfam.org/) and PROSITE (http://prosite.expasy.org/) for domain identification analysis. The non-photosynthetic species was assembled using orthologous sequences from humans, yeast, *M. brevicolis* and *N. gruberi*. Raw outputs from each analysis can be found in Additional file 3. To summarize the information about absolute proteins size, as protein domain prevalence and domain location, we plotted for every group of proteins the median protein size as a black horizontal bar (Figs 3 and 4). For every protein, the domain information was then scaled according to the median protein size of its group. This information was then converted into colored histograms, shown on top of the black bars, indicating the frequency and location of each protein function for every group of proteins. To reduce noise, domains that were detected in less than 5% of protein sequences of a protein family within a species type were not displayed.

### Gene expression and statistical analyses

Statistical comparison of each species type protein family complements was performed a non-parametric Kruskal-Wallis test in SPSS (Microsoft). Comparisons between the protein family complement of each photosynthetic eukaryote KDAC and KAT-family and the corresponding non-photosynthetic class are shown. Comparisons between each photosynthetic eukaryote species types are described in Additional file 2: Figure S1 and S2. Ortholog gene expression correlation values were obtained using Arabidopsis KDAC and KAT gene identifiers submitted to the online tool ExpressoLog ([39]; http://bar.utoronto.ca/expressolog_treeviewer/cgi-bin/expressolog_treeviewer.cgi).

### Phylogenetic tree inference

Phylogenetic tree inference by both Maximum Likelihood and Bayesian methods were performed as previously described using an LG substitution model [73]. Representative tree topologies for each gene family are depicted, with support values given for each method provided at key branch points. For PhyloBayes (Bayesian method) branch support represents the posterior probability (max value = 1.00). For the maximum likelihood method (PhyML), branch support represents a Bayesian-like transformation of the approximate likelihood ratio test value (max value = 1.00). The PhyloBayes analysis was performed using the CIPRES science gateway (https://www.phylo.org/). Phylogenetic tree visualization was performed initially in FigTree (http://tree.bio.ed.ac.uk/software/figtree/), then was exported and visualized using iTOL (http://itol.embl.de/). The taxonomic tree in Fig. 1 was constructed using phyloT (http://phylot.biobyte.de/), exported using the Newick file format and edited using FigTree (http://tree.bio.ed.ac.uk/software/figtree/).

### Subcellular localization prediction

Because the photosynthetic organisms we examined were diverse, we used five different in silico subcellular prediction algorithms to infer a consensus subcellular localization for each KDAC and KAT ortholog. These programs included WoLF pSORT ([79]; https://wolfpsort.hgc.jp/), TargetP ([80]; http://www.cbs.dtu.dk/services/TargetP/), SLP-Local ([81]; http://sunflower.kuicr.kyoto-u.ac.jp/~smatsuda/slplocal.html), PRedSL ([82]; http://aias.biol.uoa.gr/PredSL/input.html) and PREDOTAR ([83]; https://urgi.versailles.inra.fr/Tools/Predotar). Subsequent analysis in some cases was performed using MITOPROT ([84]; ihg.gsf.de/ihg/mitoprot.html) and ChloroP ([85]; http://www.cbs.dtu.dk/services/ChloroP/). A single subcellular location is denoted when 3/5 prediction programs indicate a single location. Alternatively, primary and secondary subcellular locations are denoted. Protein sequences without an indicated subcellular prediction lack an annotated N-terminal methionine (M). A complete output from each prediction program for each protein sequence is available in Additional file 5.

### Promoter element analysis

Known promoter elements were downloaded from AtCisDB (http://arabidopsis.med.ohio-state.edu/AtcisDB/) and PLACE (http://ppdb.agr.gifu-u.ac.jp/ppdb/cgi-bin/index.cgi/) (Additional file 4). Gene location files (BioMart) and genome sequence files were downloaded from http://www.phytozome.org v11 (http://www.phytozome.org) and used to collect promoter regions of 2000 bp length, upstream of all analyzed genes. If the 5′ end was closer than 2000 bp to the end of a scaffold or chromosome, the remaining sequence was used as promoter region. Promoter regions were subsequently searched for exact, non-overlapping matches for all promoter elements (Additional file 4), tracking both number of occurrences and starting position of the individual occurrences of every element in the promoter regions of every gene. Hypergeometric testing was performed to assess enrichment of promoter elements in protein families of every species individually. Candidates genes enriched in a promoter element were selected using a 0.05 *p*-value cutoff (Additional file 4). Candidate promoter elements with a presence in 50% of species examined were further analyzed for conservation across species types in relation to their annotated function.

## Additional files

Additional file 1: Tabulation of KDAC and KAT-family members from across photosynthetic eukaryotes. (XLSX 18 kb)

Additional file 2: Supplemental Figures S1–S8. (PDF 25600 kb)

Additional file 3: All protein sequences used as well as PFAM and ProSITE domain analysis raw output. (XLSX 10502 kb)

Additional file 4: All promoter element analysis data: raw, processed and summary. (XLSX 6425 kb)

Additional file 5: All in silico subcellular localization prediction raw data. (XLSX 516 kb)

## Abbreviations

CBP: p300/CREB binding protein
GNAT: GCN5/PCAF-related N-acetyltransferases
HDA/RPD3: Histone deacetylase
HDT: HD2-tuin
KAT: Lysine acetyltransferase
KDAC: Lysine deacetylase
MYST: MOZ, YBF2, SAS2, and TIP60
SRT: Sirtuin
TAF_II_250: TATA binding protein-associated factors
