# Supplementary material for: Genome-scale analysis of regulatory protein acetylation enzymes from photosynthetic eukaryotes
Source: BMC Genomics. 2017 Jul 5;18:514. doi: 10.1186/s12864-017-3894-0 (PMC5499015; doi:10.1186/s12864-017-3894-0)
Supplement: Supplementary file 2 — Supplemental Figures S1–S8. (PDF 25600 kb) [file 12864_2017_3894_MOESM2_ESM.pdf]

Additional File 2 - Introduction

Additional file 2: Figure S1

Differences in KDAC and KAT mean encoded gene number across classes of photosynthetic and non-photosynthetic (Non-PS) eukaryotes. Stars (red) denote non-parametric Kruskal-Wallis derived significance relative to non-photosynthetic Eukaryote genes of the same family (<0.05). Grey bars from left to right in each series depict Non-PS eukaryotes, Heterokonts, Glaucophytes/Algae, Moss/Bryophytes, Monocots and Dicots.

Additional file 2: Figure S2

Complete non-parametric Kruskal-Wallis test for significant differences in KDAC-family size between species types.

Additional file 2: Figure S3

Complete non-parametric Kruskal-Wallis test for significant differences in KAT-family size between species types.

Additional file 2: Figure S4

Expression correlation analysis of KDAC and KAT orthologs from A. thaliana (At), P. trichocarpa (Pt), and O. sativa (Os). Using Expressolog (http://bar.utoronto.ca; [39]), AtKDAC and AtKAT gene expression profiles were correlated with corresponding HMM identified orthologs. Gene expression and how correlation values are derived can be found in [39]. The correlation score is depicted ranging from 1 (red) to −1 (blue).

Additional file 2: Figure S5

Consensus phylogenetic tree and subcellular localization information for HDT-family KDACs from across photosynthetic and select non-photosynthetic eukaryotes. Phylogenetic tree inference and subcellular localization information was performed as outlined in the Materials and Methods. Key nodes are labelled with branch support values from 2 phylogenetic inference programs: PhyML and PhyloBayes. Node A: (0.88/0.98); Node B: (0.91/0.85); Node C: (0.94/0.99); Node D: (0.93/0.96). Consensus subcellular localization information was derived from 5 prediction

algorithms. Different species types and subcellular localizations are shown. Proteins without a known localization have no demarcation. All sequences used in phylogenetic tree generation are listed in Additional file 3, while compiled in silico subcellular localization data can be found in Additional file 5.

Additional file: Figure S6

Consensus phylogenetic tree and subcellular localization information for MYST-family KATs from across photosynthetic and select non-photosynthetic eukaryotes. Phylogenetic tree inference and subcellular localization information was performed as outlined in the Materials and Methods. Key nodes are labelled with branch support values from 2 phylogenetic inference programs: PhyML and PhyloBayes. Node A: (0.7/0.5); Node B: (0.83/0.75); Node C: (0.76/0.86); Node D: (0.99/0.86); Node E: (0.98/0.80). Consensus subcellular localization information was derived from 5 prediction algorithms. Different species types and subcellular localizations are shown. Proteins without a known localization have no demarcation. All sequences used in phylogenetic tree generation are listed in Additional file 3, while compiled in silico subcellular localization data can be found in Additional file 5.

Additional file 2: Figure S7

Consensus phylogenetic tree and subcellular localization information for $TAF_{II}250$-family KATs from across photosynthetic and select non-photosynthetic eukaryotes. Phylogenetic tree inference and subcellular localization information was performed as outlined in the Materials and Methods. Key nodes are labelled with branch support values from 2 phylogenetic inference programs: PhyML and PhyloBayes. Node A: (0.84/0.99); Node B: (0.8/0.93); Node C: (0.8/0.94); Node D: (1.0/1.0); Node E: (1.0/1.0). Consensus subcellular localization information was derived from 5 prediction algorithms. Different species types and subcellular localizations are shown. Proteins without a known localization have no demarcation. All sequences used in phylogenetic tree generation are listed in Additional file 3, while compiled in silico subcellular localization data can be found in Additional file 5.

Additional file 2: Figure S8

Alignment of KIX-like domain containing PS eukaryote CBP-family KATs. Photosynthetic eukaryote CBP KATs were examined for the presence of an-terminal KIX domain. Neither PFAM nor ProSITE resolved a KIX domain in photosynthetic eukaryote CBP KATs despite its detection in non-photosynthetic eukaryote CBP KATs (Fig. 3). Highlighted (yellow) are three regions corresponding to the conserved alpha helices comprising the KIX domain of metazoan CBP KATs. HsCBP (Q92793) was used as a reference for alignment of photosynthetic eukaryote CBP KATs. This revealed that photosynthetic eukaryote CBP KATs maintain a putative n-terminal KIX-like domain which is divergent from those previously identified in metazoans.

Fig. S1

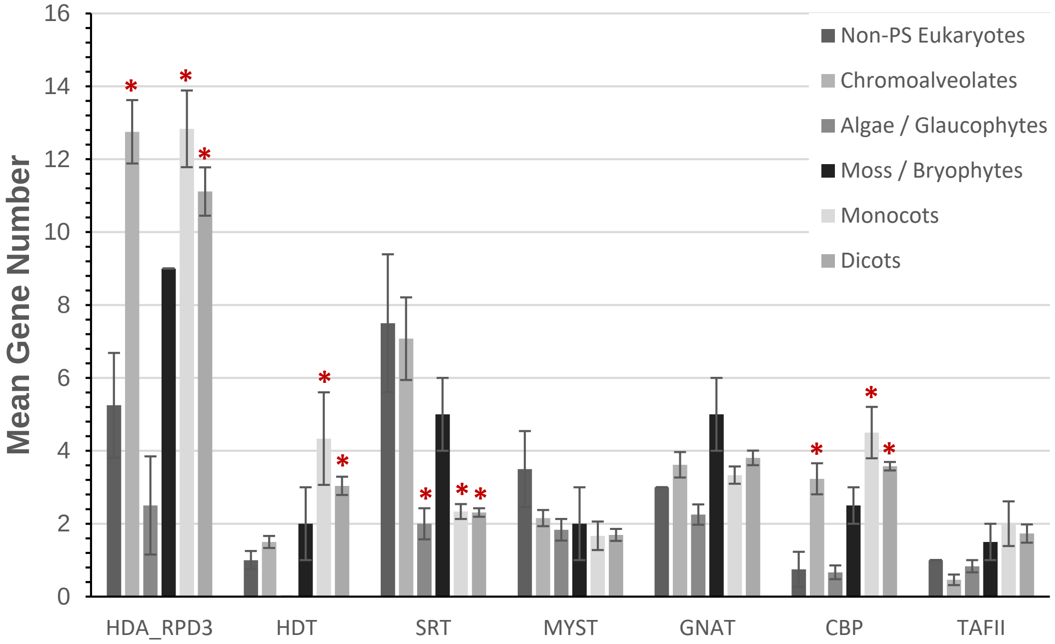
Mean Gene Number
16
14
12
10
8
6
4
2
0
Non-PS Eukaryotes
Chromoalveolates
Algae / Glaucophytes
Moss / Bryophytes
Monocots
Dicots
HDA_RPD3
HDT
SRT
MYST
GNAT
CBP
TAFII

Fig. S2

Lysine Deacetylase (KDACs)

HDA-family

Each node shows the sample average rank of V1.

Sample1-Sample2	Test Statistic	Std. Error	Std. Test Statistic	Sig.	Adj.Sig.
1.00-3.00	-11.667	10.525	-1.108	.268	1.000
1.00-4.00	-17.000	15.788	-1.077	.282	1.000
1.00-2.00	-20.731	10.424	-1.989	.047	.701
1.00-6.00	-25.173	9.791	-2.571	.010	.152
1.00-5.00	-37.500	11.768	-3.187	.001	.022
3.00-4.00	-5.333	13.924	-.383	.702	1.000
3.00-2.00	9.064	7.298	1.242	.214	1.000
3.00-6.00	-13.506	6.362	-2.123	.034	.506
3.00-5.00	-25.833	9.115	-2.834	.005	.069
4.00-2.00	3.731	13.847	.269	.788	1.000
4.00-6.00	-8.173	13.378	-.611	.541	1.000
4.00-5.00	-20.500	14.885	-1.377	.168	1.000
2.00-6.00	-4.442	6.193	-.717	.473	1.000
2.00-5.00	-16.769	8.998	-1.864	.062	.935
6.00-5.00	12.327	8.257	1.493	.135	1.000

Each row tests the null hypothesis that the Sample 1 and Sample 2 distributions are the same.
Asymptotic significances (2-sided tests) are displayed. The significance level is .05.

HDT-family

Each node shows the sample average rank of V1.

Sample1-Sample2	Test Statistic	Std. Error	Std. Test Statistic	Sig.	Adj.Sig.
3.00-2.00	3.038	7.041	.432	.666	1.000
3.00-1.00	4.000	10.155	.394	.694	1.000
3.00-4.00	-24.500	13.433	-1.824	.068	1.000
3.00-6.00	-32.077	6.138	-5.226	.000	.000
3.00-5.00	-37.833	8.794	-4.302	.000	.000
2.00-1.00	.962	10.056	.096	.924	1.000
2.00-4.00	-21.462	13.359	-1.606	.108	1.000
2.00-6.00	-29.038	5.974	-4.860	.000	.000
2.00-5.00	-34.795	8.681	-4.008	.000	.001
1.00-4.00	-20.500	15.232	-1.346	.178	1.000
1.00-6.00	-28.077	9.446	-2.972	.003	.044
1.00-5.00	-33.833	11.353	-2.980	.003	.043
4.00-6.00	-7.577	12.906	-.587	.557	1.000
4.00-5.00	-13.333	14.361	-.928	.353	1.000
6.00-5.00	5.756	7.966	.723	.470	1.000

Each row tests the null hypothesis that the Sample 1 and Sample 2 distributions are the same.
Asymptotic significances (2-sided tests) are displayed. The significance level is .05.

SRT-family

Each node shows the sample average rank of V1.

Sample1-Sample2	Test Statistic	Std. Error	Std. Test Statistic	Sig.	Adj.Sig.
3.00-6.00	-3.115	6.126	-.509	.611	1.000
3.00-5.00	-5.083	8.777	-.579	.562	1.000
3.00-4.00	-29.000	13.407	-2.163	.031	.458
3.00-2.00	30.981	7.027	4.409	.000	.000
3.00-1.00	34.125	10.135	3.367	.001	.011
6.00-5.00	1.968	7.950	.248	.805	1.000
6.00-4.00	25.885	12.881	2.009	.044	.667
6.00-2.00	27.865	5.963	4.673	.000	.000
6.00-1.00	31.010	9.428	3.289	.001	.015
5.00-4.00	23.917	14.333	1.669	.095	1.000
5.00-2.00	25.897	8.664	2.989	.003	.042
5.00-1.00	29.042	11.331	2.563	.010	.156
4.00-2.00	1.981	13.333	.149	.882	1.000
4.00-1.00	5.125	15.202	.337	.736	1.000
2.00-1.00	3.144	10.037	.313	.754	1.000

Each row tests the null hypothesis that the Sample 1 and Sample 2 distributions are the same.
Asymptotic significances (2-sided tests) are displayed. The significance level is .05.

1. Non-photosynthetic Eukaryotes
2. Heterokonts
3. Algae
4. Moss / Bryophytes
5. Monocots
6. Dicots

Fig. S3

Lysine Acetyltransferase (KATs)

CBP-family

Each node shows the sample average rank of V1.

Sample1-Sample2	Test Statistic	Std. Error	Std. Test Statistic	Sig.	Adj.Sig.
3.00-1.00	.875	10.416	.084	.933	1.000
3.00-4.00	-19.000	13.779	-1.379	.168	1.000
3.00-2.00	27.058	7.222	3.747	.000	.003
3.00-6.00	-28.500	6.296	-4.527	.000	.000
3.00-5.00	-39.333	9.020	-4.361	.000	.000
1.00-4.00	-18.125	15.624	-1.160	.246	1.000
1.00-2.00	-26.183	10.315	-2.538	.011	.167
1.00-6.00	-27.625	9.689	-2.851	.004	.065
1.00-5.00	-38.458	11.645	-3.302	.001	.014
4.00-2.00	8.058	13.703	.588	.557	1.000
4.00-6.00	-9.500	13.238	-.718	.473	1.000
4.00-5.00	-20.333	14.730	-1.380	.167	1.000
2.00-6.00	-1.442	6.128	-.235	.814	1.000
2.00-5.00	-12.276	8.904	-1.379	.168	1.000
6.00-5.00	10.833	8.171	1.326	.185	1.000

Each row tests the null hypothesis that the Sample 1 and Sample 2 distributions are the same.
Asymptotic significances (2-sided tests) are displayed. The significance level is .05.

GNAT-family

Each node shows the sample average rank of V1.

Sample1-Sample2	Test Statistic	Std. Error	Std. Test Statistic	Sig.	Adj.Sig.
3.00-1.00	8.542	9.983	.856	.392	1.000
3.00-5.00	-16.542	8.646	-1.913	.056	.836
3.00-6.00	-19.349	6.034	-3.206	.001	.020
3.00-2.00	20.657	6.922	2.984	.003	.043
3.00-4.00	-37.042	13.206	-2.805	.005	.076
1.00-5.00	-8.000	11.161	-.717	.474	1.000
1.00-6.00	-10.808	9.287	-1.164	.245	1.000
1.00-2.00	-12.115	9.887	-1.225	.220	1.000
1.00-4.00	-28.500	14.975	-1.903	.057	.855
5.00-6.00	-2.808	7.831	-.359	.720	1.000
5.00-2.00	4.115	8.534	.482	.630	1.000
5.00-4.00	20.500	14.118	1.452	.146	1.000
6.00-2.00	1.308	5.874	.223	.824	1.000
6.00-4.00	17.692	12.688	1.394	.163	1.000
2.00-4.00	-16.385	13.134	-1.248	.212	1.000

Each row tests the null hypothesis that the Sample 1 and Sample 2 distributions are the same.
Asymptotic significances (2-sided tests) are displayed. The significance level is .05.

TAF_{II}250-family

Each node shows the sample average rank of V1.

Sample1-Sample2	Test Statistic	Std. Error	Std. Test Statistic	Sig.	Adj.Sig.
2.00-3.00	-8.571	6.588	-1.301	.193	1.000
2.00-1.00	12.654	9.409	1.345	.179	1.000
2.00-6.00	-22.942	5.590	-4.104	.000	.001
2.00-4.00	-23.404	12.499	-1.872	.061	.917
2.00-5.00	-26.321	8.122	-3.241	.001	.018
3.00-1.00	4.083	9.501	.430	.667	1.000
3.00-6.00	-14.372	5.743	-2.502	.012	.185
3.00-4.00	-14.833	12.568	-1.180	.238	1.000
3.00-5.00	-17.750	8.228	-2.157	.031	.465
1.00-6.00	-10.288	8.838	-1.164	.244	1.000
1.00-4.00	-10.750	14.251	-.754	.451	1.000
1.00-5.00	-13.667	10.622	-1.287	.198	1.000
6.00-4.00	.462	12.075	.038	.970	1.000
6.00-5.00	3.378	7.453	.453	.650	1.000
4.00-5.00	-2.917	13.436	-.217	.828	1.000

Each row tests the null hypothesis that the Sample 1 and Sample 2 distributions are the same.
Asymptotic significances (2-sided tests) are displayed. The significance level is .05.

MYST-family

No significant changes amongst any groups

1. Non-photosynthetic Eukaryotes
2. Heterokonts
3. Algae
4. Moss / Bryophytes
5. Monocots
6. Dicots

Fig. S4

KDACs

HDA-FAMILY	Ortholog	Development	Stress
AtHDAj (AtHDA2)	PtHDAj	0.5	0.2
	OsHDAm	0.5	1
AtHDAi / h (AtHDA5 / 18)	PtHDAg	1	0.7
	OsHDAi	0.5	-0.5
AtHDAd / c (AtHDA6 / 7)	PtHDAc	0.8	0
	PtHDAe	1	0.4
	OsHDAa	1	1
AtHDAf (AtHDA8)	PtHDAf	0.7	0.4
	PtHDAl	n/a	n/a
	OsHDAq	n/a	n/a
	OsHDAr	n/a	n/a
AtHDAb (AtHDA9)	PtHDAd	-0.4	-0.4
	OsHDAb	0.7	0.4
AtHDAe (AtHDA14)	PtHDAh	0.7	-0.2
	PtHDAk	n/a	n/a
	OsHDAk	1	-0.2
AtHDAg (AtHDA15)	PtHDAi	1	1
	OsHDAj	0.3	0.1
AtHDAa (AtHDA19)	PtHDAa	-0.4	-0.4
	PtHDAb	1	-0.1
	OsHDAd	0.3	n/a
	OsHDAe	0	1
	OsHDAf	0.6	1

HDT-FAMILY	Ortholog	Develop	Stress
AtHDTc	PtHDTc	0.5	0.3
	OsHDTc	0.7	0.3

*No probes for remainder

SRT-FAMILY	Ortholog	Develop	Stress
AtSRTa (AtSRT1)	PtSRTa	0.6	1
	OsSRTa	1	0
AtSRTb (AtSRT2)	PtSRTb	0.5	0.3
	OsSRTb	0.7	0.3

KATs

MYST-FAMILY	Ortholog	Development	Stress
AtMYSTa	PtMYSTa	0.6	0.5
	PtMYSTb	-0.3	-0.7
	OsMYSTa	1	1
AtMYSTb	PtMYSTa	0.2	-0.3
	PtMYSTb	1	0.8
	OsMYSTa	1	-0.3

GNAT-FAMILY	Ortholog	Development	Stress
AtGNATa (HAG1 / GCN5-like)	PtGNATa	-0.5	0
	PtGNATb	-0.3	-0.3
	OsGNATa	0.6	-0.6
AtGNATb (HAG2 / HAT1-like)	PtGNATc	1	0.8
	OsGNATb	0.8	1
AtGNATc (HAG3 / ELP3-like)	PtGNATd	1	0.4
	PtGNATe	1	0.4
	OsGNATc	0.9	0.3

CBP-FAMILY	Ortholog	Development	Stress
AtCBPa	PtCBPc	0.4	0.4
	OsCBPa	1	-0.7
	OsCBPb	1	-0.7
	OsCBPc	0.5	1
AtCBPb	PtCBPc	n/a	n/a
	OsCBPa	0	0.4
	OsCBPb	0.3	0.4
	OsCBPc	-0.3	-0.3
AtCBPc	PtCBPc	n/a	n/a
	OsCBPa	n/a	n/a
	OsCBPb	n/a	n/a
	OsCBPc	n/a	n/a
AtCBPd	PtCBPc	1	0.4
	OsCBPa	1	-0.7
	OsCBPb	1	-0.7
	OsCBPc	0.4	1
AtCBPe	PtCBPc	0	0.6
	OsCBPa	1	-0.7
	OsCBPb	0.8	-0.7
	OsCBPc	0.6	1

TAFII-FAMILY	Ortholog	Development	Stress
AtTAFIIa	PtTAFIIa	0.7	0.4
	OsTAFIIa	1	1

*No probes for remainder

Fig. S5

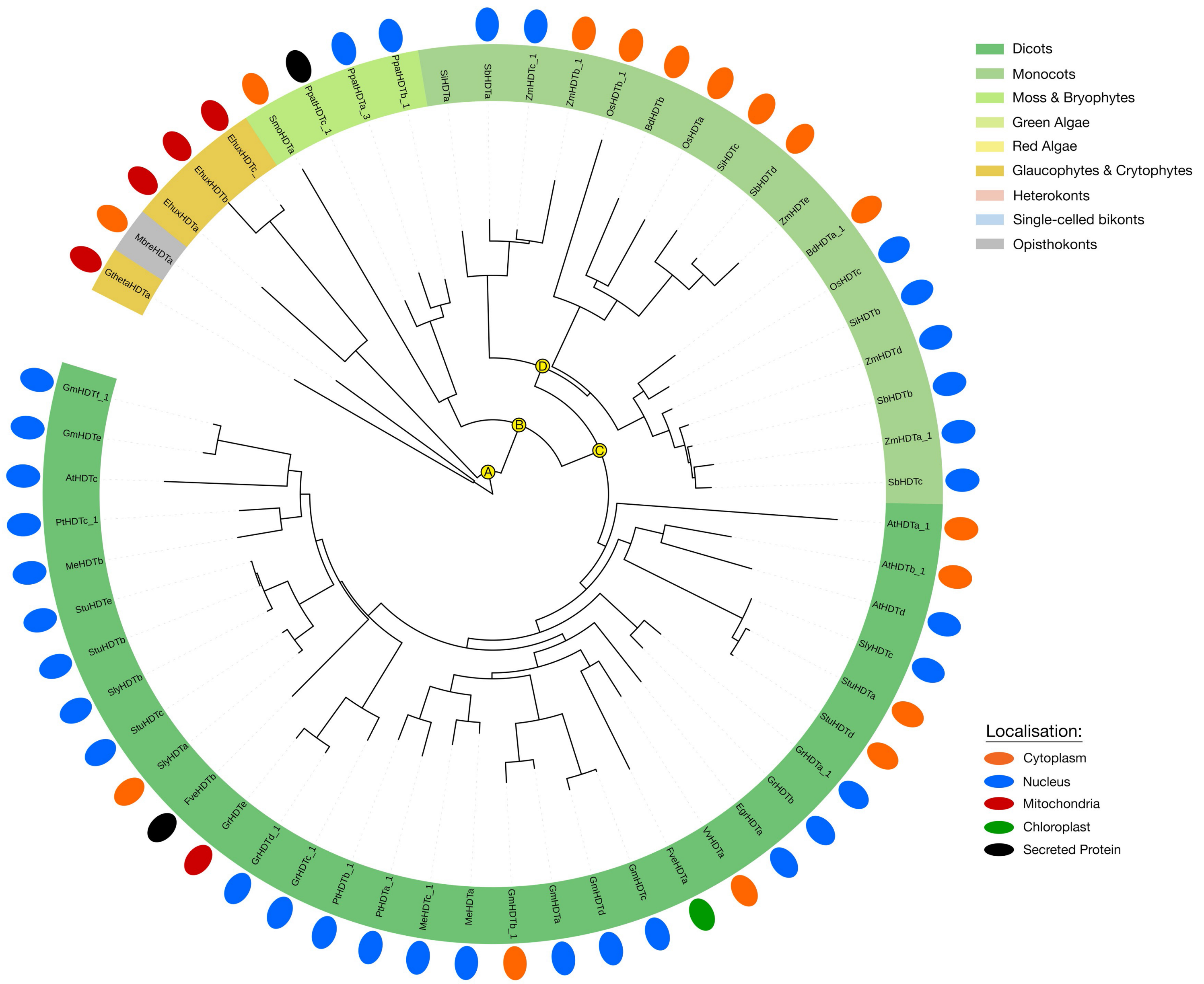

Dicots
Monocots
Moss & Bryophytes
Green Algae
Red Algae
Glaucophytes & Crytophytes
Heterokonts
Single-celled bikonts
Opisthokonts
Localisation:
Cytoplasm
Nucleus
Mitochondria
Chloroplast
Secreted Protein

Fig. S6

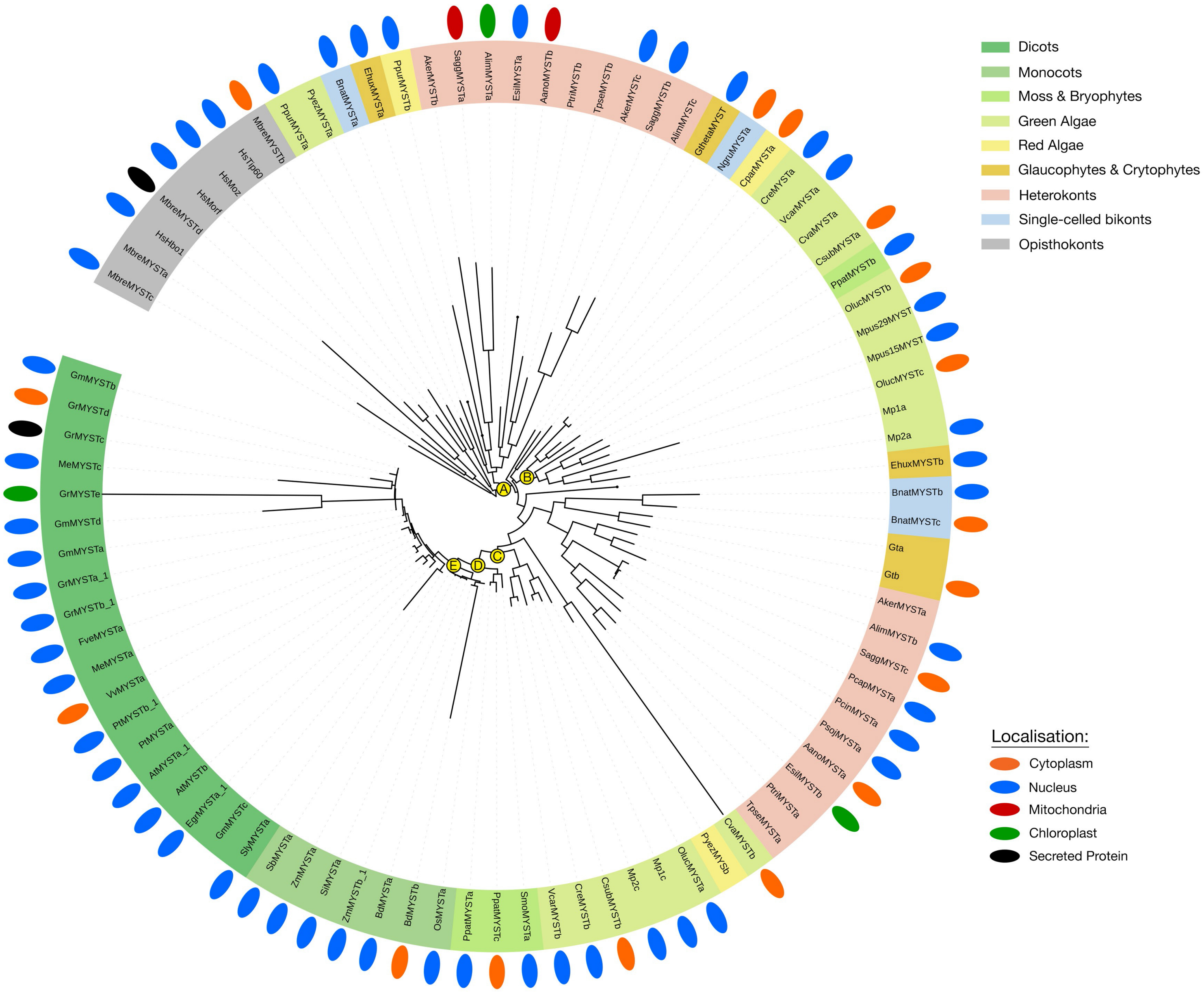

Dicots
Monocots
Moss & Bryophytes
Green Algae
Red Algae
Glaucophytes & Crytophytes
Heterokonts
Single-celled bikonts
Opisthokonts
Localisation:
Cytoplasm
Nucleus
Mitochondria
Chloroplast
Secreted Protein

Fig. S7

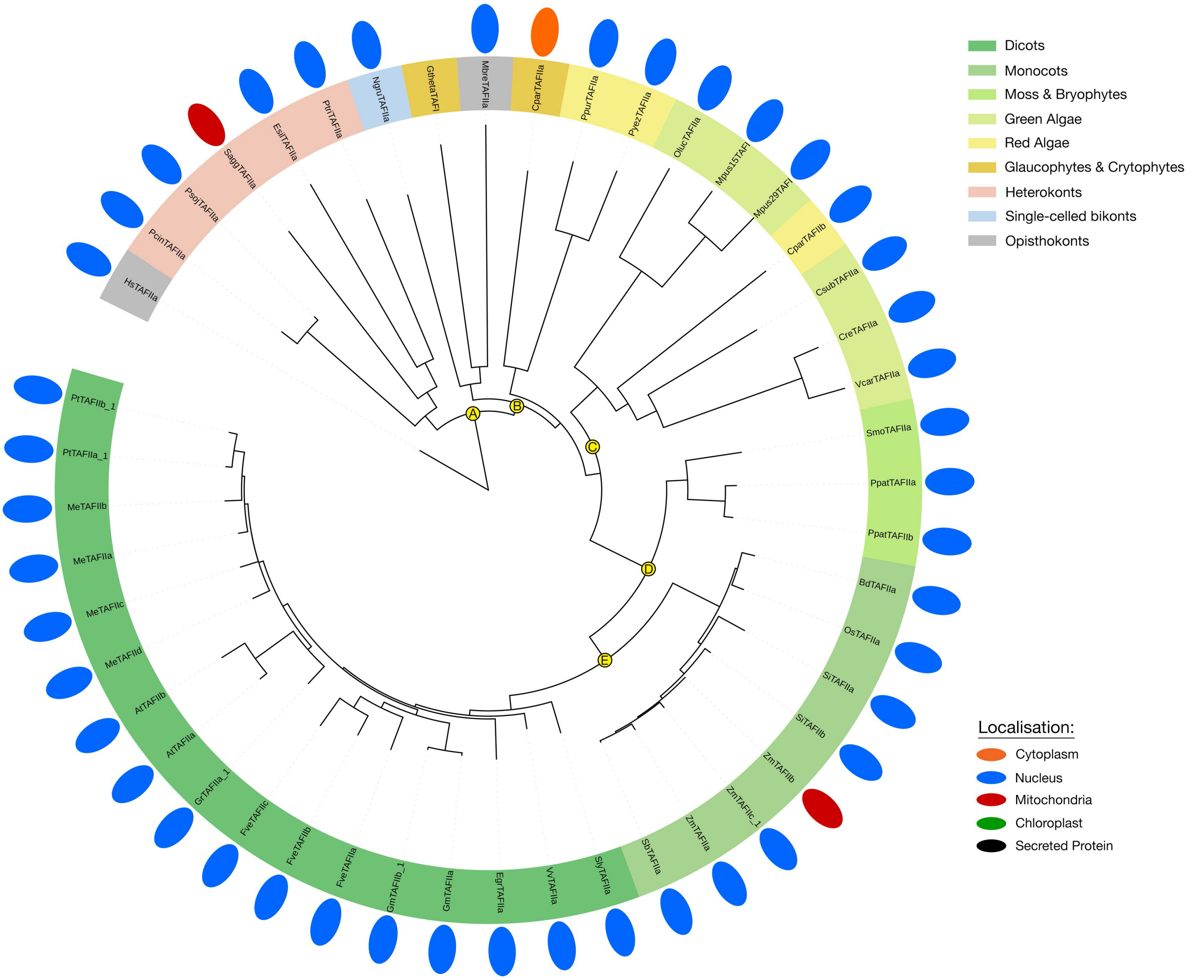

HsTAFIIa
PcinTAFIIa
PsojTAFIIa
SaggTAFIIa
EsiTAFIIa
PinTAFIIa
NgruTAFIIa
GthetaTAFI
MbreTAFIIa
CparTAFIIa
PpurTAFIIa
PyezTAFIIa
OlucTAFIIa
Mpus15TAFI
Mpus29TAFI
CparTAFIIb
CsubTAFIIa
CreTAFIIa
VcarTAFIIa
SmoTAFIIa
PpatTAFIIa
PpatTAFIIb
BdTAFIIa
OsTAFIIa
SiTAFIIa
SiTAFIIb
ZmTAFIIb
ZmTAFIIc_1
ZmTAFIIa
SbTAFIIa
SlyTAFIIa
VvTAFIIa
EgTAFIIa
GmTAFIIa
GmTAFIIb_1
FveTAFIIa
FveTAFIIb
FveTAFIIc
GrTAFIIa_1
AtTAFIIa
AtTAFIIb
MeTAFIId
MeTAFIIc
MeTAFIIa
MeTAFIIb
PtTAFIIa_1
PtTAFIIb_1
A
B
C
D
E
Dicots
Monocots
Moss & Bryophytes
Green Algae
Red Algae
Glaucophytes & Crytophytes
Heterokonts
Single-celled bikonts
Opisthokonts
Localisation:
Cytoplasm
Nucleus
Mitochondria
Chloroplast
Secreted Protein

Fig. S8

```
                                       α1                                      α2                           α3
                         *         20         *        40         *        60         *        80
HsCBP_KIX   : VRKGWH-EHVTQDLRSHLVH-KLVQAIFPTPDPAALKD-RRMENLVAYAKKVEGDMYESANSRDEYYHLLAEKIYKIQKELEE- : 80
MbreCBPa    : --QARASRDSHCKAQV-SRAQVSRAQVSR--------SRAR--AFKD--RVFKDRVFKANPSKDNFKDSLSDSPRCKQSLVEA- : 68
AtCBPa_1    : PSRVSPVDNDILKLRQ-AMRIRIFNILQQKQPSPADEASKA--KYMDVARRLEEGLFKIANTKEDYVNP--STLEPRASLIKG- : 78
AtCBPd_1    : PSRVGAMDHDIMKLRQ-YMQTLVFNMLQQRQPSPADAASKA--KYMDVARRLEEGLFKMAVTKEDYMNR--STLESRTSLIKG- : 78
BdCBPb      : -----GMDHEFISMRR-TMLERIADIFKKTRG---LTKTNQ--QLMHLVKQLEVFMYKKHPTKAGYYEMLKGSIEVHAEAYKA- : 72
BdCBPd      : ----VGNDPHFVMLRN-SMREKIFEYIGRKQS---SAEWRR--RLPELARRLEEILFRKFPNKTEYYSMMKAPVEPQQFAIKT- : 73
OsCBPb      : -----GLDTQFLLMRN-TMRDRIFEYIGRKQS---STDWRR--RLPELAKRLEEILYRKFLNKADYLNMMRGPVEPQQFAIKT- : 72
SbCBPa      : -----GTDQHFVMLRT-TMREKIFEYIGRKQS---SAEWRK--RLPELAKRLEEILYRKFPNKNDYYNMMKGPVEPQQFAIKT- : 72
ZmCBPd      : -----GTDQQFVMFRT-TMREKIFNYIGRKQS---SAEWRK--RLPELAKRLEEILYRKFPNKTDYYNMMKGPVEPQQFAIKT- : 72
SiCBPb      : -----GTDQQFVMLRT-TMRDRIFECLGRKQL---SAEWRK--RLPELAKRLEEMLFRKFPNRNEYYNMMNGPVEPQQFAIKT- : 72
ZmCBPb      : -----GMDQQFVMLRT-AMRDKISDYIGRKQS---PGDWRK--RLPELAKRLEEILYRKFPNKHEYYNMMKGPVEPQQFAIKT- : 72
MeCBPa_1    : SPNMFTMDPELHRARI-FMREKIFAIILQRQPQPVSEPQKQ--KFKDIAKRLEEGLFKAAQSKEDYMNL--NTLESRSSLIKR- : 78
PtCBPa      : SVNVYNAEPELHRYRL-YMQQKIFSIILQKQSQPVGDQQKQ--RFKEFAKRLEEGLFKAAQTKDDYLNM--NTLESRSSLLKR- : 78
PtCBPb_1    : PANMYSIDPELRRAR-NYIHHKIFEIIMRRHSQPVDDTQKQ--KFKGIAKRLEEGLFKAAQTKEDYLNL--NTLESRSSLIKR- : 78
GrCBPa      : PQNTLNMVPDIIRTR-EYMQGQIIDFLKSRYQRPITETSML--KLRDFARHLEEGLFKIARTKDDYTNL--STLEHRQILMR-- : 77
GrCBPb_1    : PHTMSNMDPELHRTR-EYMRGKIIDVLKLRNQLPITEASMM--KFRDFARRLEEGLFKIAHTKEDYTNL--STLEHRQILLR-- : 77
GrCBPc_1    : PHNMLNMDPDLMRTR-DYMRGEIIKFLKSRQQHPITEASMI--KFQDFARRLEEGLFKIARTKEDYTNL--STLEQRQILIR-- : 77
GrCBPe      : PHNTLNMDTDLMRIRE-FMRGKITEILKLRNQHPITEASMM--KFRDFARRLEEGLFKIARTKEDYANL--NTLEQRQILIK-- : 77
GmCBPa_1    : PRSTINMDPDFLRART-FIHDKIFDMLLQRQQQPVTDVQRK--KLKDLAKRLEEGMLKAALSKEDYMNL--DTLESRSNFLRR- : 78
GmCBPe_1    : PRSTINMDPEFLRART-FIQEKIFDMLLQRQQLPVTDVQRR--KLKDLANRLEEGMLKAALSKEDYMNL--DTLESRSNFLRR- : 78
GmCBPd_1    : --VCTTMDPEFLRARS-LTLEKIYNILLQRYQHPVTEAHRR--KVKDLAKRLEEGMFKTAISKEDYMNL--DTLESRSNFLRR- : 76
FveCBPa     : PRAMSSMDPELIRARQ-FMQEKICHVIQQR---PLPQMNEK--KFRDIVKRLEEGLLRSAVTKEDYMNL--DTLESRHNLIKR- : 75
SlyCBPa     : --TIPNMEFELVKVRR-TISRRIYEYLIRRQQQQIQEAQHQ--RIVDLVKRLEESLFKSASTKEEYMDL--STLENRLSVIKR- : 76
SlyCBPd     : --NVLNMEPDFSRARI-FISNKIYDYLM--QRQQSHEKPPK--KVMDIVKRLEEGLFKSASSKEEYLNQ--ATLENRHVLIKS- : 74
AtCBPe      : PNMVNGISQDTLLLRQ-EMLNRTYAWLQQRQPSKTDDASKA--KLSEVAKRLESAMWRTATSKEDYLDF--RSFDVRESTLKQ- : 78
AtCBPc      : ----------IKKRRT-ALRNRIYAIVRHKQQQRVDDATQR--ALLEATRMIEEELLKSSRSFEEYFDL--RTFDARRTILQQ- : 68
FveCBPb     : ---DWRKGPGVSWLRT-LVRRKILERFLGC-------SAKG--NLASFVLGIETQLFMEATSEDDYVNE--QTINDRLSAVQQ- : 68
GmCBPc      : PNHDWRKDPEISQCRN-LIKNDISTILHSRDQGQASV----------LANFLENRLFVEAASKDEYINR--QTIMQRDTQLKK- : 70
AtCBPb      : P------------RK-RTRDLMPKFLNTE----FDE----------FNQRLNNLPAESNVTSDEDAQF----LESR------- : 45
BdCBPa      : ---LDNMDSDTNPVRF-MIQQRILHCLKER-------WNPH--CLVELSKQIDEQLYKDA-SKVQYMDF--DTIEERNAVLST- : 67
OsCBPa_1    : ---LDNMDQDTSVVRN-IIHRKIVEYLNER-------FDLS--FLMEIGKCIDRHLFEKADSKIKYMDL--ETLRTRNAIVNS- : 68
SbCBPb      : ---LDNMNQNTSPIRA-IIIEKIEACLRNM-------LCPD--SCLKASRIIDNMLYKNA-PQLHYLDL--ETVEGRNDLLRS- : 67
ZmCBPa      : ---LDNMDQGTSPVRA-VIIKRIETCLRNR-------LCPD--SLLKASEIIDNLLYKID-PQPQYMDL--DTIEARNDLLRS- : 67
SiCBPd      : ---PDNMDQSTSPIRG-IIIQKIASCLKKR-------TSPD--YLLKFSRKIDECLYKRA-PKVHYMDL--STLEVRNALLNS- : 67
ZmCBPc      : ---LDNMDQNTSPVRR-LIMNKIEACLKKS-------FRPY--FLRFPSRIIDSILYETA-P-------------------- : 48
OsCBPc_1    : ---HASADPELMNLRI-RMTNRIWELLSRE-------TRPR--KLVSLAKRFEAVIYKKNPNKAAYYSILNGEIFPHQHALST- : 70
MeCBPb      : ---DWRKNPRISCFRD-LVREKIAESFE---------EENH--QVQELVNVLENKLFAEAASEEEYMNL--DVLRERQNLFQK- : 66
PpatCBPa    : PRWQSEQDTSL---RR-HIVENILQLFKNRKPH-VTPEWQQ--KLPDFVKRLEEALYKNAPSKEEYADL--KTLEQRQSVARR- : 74
CreCBPa     : ---SWQSEADTAD-RQ-RMTDHIYHIFQSKSQQPYNN------KLPEFVKRLEDGLYRSAKSKDEYTDM--NTLEQRHDVAKR- : 70
Mpus15CBPa  : ---TWQSDADIPQ-RK-VLIQHILKLFRQR---PSETEWQQ--KLPDFVLRLEEAVYRGARSKEEYCNE--ATLEARQAVAR-- : 70
Mpus29CBPb  : ---TWQSDDDIPQ-RK-VLIQHILRLFQQR---PSETEWAQ--KLPDFVLRLEEAVYRGARTKEEYCNE--QTLEARQAVAR-- : 70
PtriCBPb    : P---WQSDKDYQERRK-MI-AKIVHLLQQR-------EWLK--KLPQMAKRLEESLYRTATSFEEYNDA--NTLKHRQQLAVN- : 67
TpseCBPa    : PNF-WQSDRDMHHRRE-MI-QHIVKLLKQK-------EWLT--KLPQMVKQLEVSLYRSAPSFEAYSDT--NTLKHRQQLAME- : 69
PcinCBPa    : P--TWQSSADQALRKE-ML-NHIMRVLQNQ-------NWLQ--KLPEMAHRLEDECYRIAASREEYANT--GTLQSRRSIVRS- : 68
PsojCBPb    : P--TWQSSADQALRKE-ML-NHIMRVLQNQ-------NWLQ--KLPEMAHRLEDECYRIAASREEYANT--STLQSRRSIVRS- : 68
PcinCBPc    : ---DWRDKDADRGARQ-QIARIILTLIMKS-----------------IARRLEHTLYMGAPSKQEYNNV--QTLKARQQLQA- : 60
PtriCBPa    : PNQTNTNHTNGGGAQQ-LMLQQQIASLQKQPRAANSLSHQQ--QLLQMSQSLHQQMLQQSHARNNMANVMLNEQQLRQNLLRA- : 80
```
